# Supplementary material for: Relationship between anaemia, coagulation parameters during pregnancy and postpartum haemorrhage at childbirth: a prospective cohort study
Source: BMJ Open. 2021 Oct 3;11(10):e050815. doi: 10.1136/bmjopen-2021-050815 (PMC8491293; doi:10.1136/bmjopen-2021-050815)

Table-S1: Traceability of Assays

Sl No	Name of test	Calibrator traceability (reference material/ reference method)	Units	Typical Calibrator value	Calibrator uncertainty of measurement
1	Haemoglobin	1:250 dilution in NCCLS2 recommended reagent for the hemiglobincyanide (cyanmethemoglobin	g/dl	12.58	1.00%
2	Hematocrit	Calculated	%	calculated	NA
3	Platelets	A 1:101 dilution is made using a 20 μL TC pipette and 2 mL of 1% filtered ammonium oxalate (CLSI/ formerly NCCLS)	thou/mm3	214.1	6.00%
4	Serum Ferritin	WHO 3rd International Standard 94/572	ng/ml	Low 5.44<br>High 953	Low 19.5<br>High 9.3
5	Haemoglobin electrophoresis	NGSP Certification for A2/F	%	HbF-6.6 % and HbA2-6.7 %	HbF- Low- NA<br>High 1.8 %<br>HbA2- Low-NA, High- 3.6 %
6	D-Dimer	Pre-calibrated	mg/L FEU	NA	NA
7	Fibrinogen, Clotting activity	WHO	mg/dl	269	2.7%
8	International Normalized Ratio (INR)	Calculated			

NGSP - National Glycohemoglobin Standardization Program; CLSI – Clinical and Laboratory Standards Institute; HbF – Fetal haemoglobin; HbA2 - Haemoglobin Subunit Alpha 2; NA - Not applicable; WHO – World Health Organisation

Nair M, *et al. BMJ Open* 2022; 11:e050815. doi: 10.1136/bmjopen-2021-050815

Table-S2: Assay Information and performance characteristics

Sl No	Name of test	System used for the analysis	Method information (supplier/ method)	Manufacturers' Analytical Range	Laboratory Reportable range	Normal Reference range	Biological variation	Uncertainty of measurement	QC material	External Quality Assurance
1	Haemoglobin	DxH -800 (Beckman Coulter, Fullerton, CA, USA)	Photometric	0.0-99.9g/dl	1.25	13.-17.0g/dl (adult male)	2.1	4.0	Coulter 6c cell control	CAP
2	Hematocrit	DxH -800 (Beckman Coulter, Fullerton, CA, USA)	Automated calculation	0.99.9	NA	40-50% (adult male)	1.9	4.0	Coulter 6c cell control	CAP
3	Platelets	DxH -800 (Beckman Coulter, Fullerton, CA, USA)	Impedance/ Coulter principle	0.7000	10-1000	150-450thou/mm3 (adult male)	2.9	6.0	Coulter 6c cell control	CAP
4	Serum Ferritin	Siemens ADVIA Centaur	Chemiluminescence Immunoassay (CLIA)	0.5 – 1650 ng/ml	<0.5, >16500		14.2	22.5	BIO-RAD	CAP PT
5	Haemoglobin electrophoresis	Variant II Hemoglobin testing system (Bio-Rad, Hercules, CA, USA)	High Performance Liquid Chromatography	HbF-1.3-44.3 % HbA2-1.6-18.7 %	HbF-1.3-99.8% HbA2-1.6-18.7 %	HbF- <1.5 % HbA2-1.5-3.5 %	HbF-5.6 % HbA2-4.4 %	HbF-10.97 % HbA2-8.62 %	BIO-RAD	CAP
6	D-Dimer	STA-R Evolution (Diagnostica Stago, Cedex, France)	Latex-enhanced immunoturbidimetry	0.22-20.0	0.22-20.0	< 0.50	10.4	20.38	Stago	CAP
7	Fibrinogen, Clotting activity	Sysmex CS5100 analyzer (Sysmex Corporation, Kobe, Japan)	Photo optical clot detection	30 -1400	50 -1200	200 - 400	13.8	27.05	Siemens	CAP
8	International Normalized Ratio (INR)	Calculated from Prothrombin time measured by Photo-optical clot Detection on Sysmex CS5100 analyzer (Sysmex Corporation, Kobe, Japan)	Calculated	Calculated	NA	0.9 – 1.1	NA	NA	Siemens	CAP

INR: International Normalized Ratio; NABL: National Accreditation Board for Testing and Calibration; CAP: College of American Pathologists; CAP PT - College of American Pathologists Proficiency Testing programme; HbF: Fetal haemoglobin; HbA2: Haemoglobin Subunit Alpha; QC: Quality Control; NA - Not applicable

Table-S3: Sample size calculations

Coagulation parameter	Concentration in general pregnant population Mean (SD)	Sample required for a range of expected difference in mean concentration of the blood parameters between the groups of anaemia in either direction					
		10% difference		20% difference		30% difference	
		n1	Total	n1	Total	n1	Total
D-Dimer (mg/dl)	0.11 (0.573)[7]	**514**	**1028**	129	258	58	116
Fibrinogen (mg/dl)	379 (0.78)[8]	90	180	23	46	10	20

n1 – women with haemoglobin concentration <10g/dl; n2 – women with haemoglobin concentration ≥10g/dl

Table-S4: Association of coagulation parameters with haematocrit at third trimester

**Independent variables**	**Outcome variables**	
	D-Dimer	
	Unadjusted coefficients (95% CI)	Adjusted* coefficients (95% CI)
Haematocrit (HCT)	0.99 (0.98 to 0.99)	0.99 (0.98 to 0.99)
HCT ≥30%	1 (ref)	1 (ref)
HCT <30%	1.13 (1.03 to 1.25)	1.13 (1.03 to 1.24)
	Fibrinogen	
	Unadjusted coefficients (95% CI)	Adjusted* coefficients (95% CI)
Haematocrit (HCT)	4.57 (3.37 to 5.77)	5.69 (4.51 to 6.88)
HCT ≥30%	0 (Ref)	0 (Ref)
HCT <30%	-58.3 (-75.4 to -41.1)	-68.1 (-84.8 to -51.3)
	D-Dimer/Fibrinogen ratio	
	Unadjusted coefficients (95% CI)	Adjusted* coefficients (95% CI)
Haematocrit (HCT)	0.98 (0.97 to 0.99)	0.97 (0.96 to 0.98)
HCT ≥30%	1 (ref)	1 (ref)
HCT <30%	1.35 (1.19 to 1.52)	1.39 (1.24 to 1.56)
	INR	
	Unadjusted coefficients (95% CI)	Adjusted* coefficients (95% CI)
Haematocrit (HCT)	0.99 (0.99 to 1.00)	0.99 (0.99 to 1.00)
HCT ≥30%	1 (ref)	1 (ref)
HCT <30%	1.02 (0.98 to 1.06)	1.05 (1.01 to 1.09)
	Platelets	
	Unadjusted coefficients (95% CI)	Adjusted* coefficients (95% CI)
Haematocrit (HCT)	-0.38 (-1.07 to 0.30)	-0.89 (-1.59 to -0.18)
HCT ≥30%	0 (ref)	0 (ref)
HCT <30%	4.84 (-4.93 to 14.61)	11.32 (1.42 to 21.23)

*Adjusted for gestational age, maternal age, PIH, pre-existing medical problems and hospital-code.

Nair M, *et al. BMJ Open* 2022; 11:e050815. doi: 10.1136/bmjopen-2021-050815

Figure-S1: Flow chart showing the study population

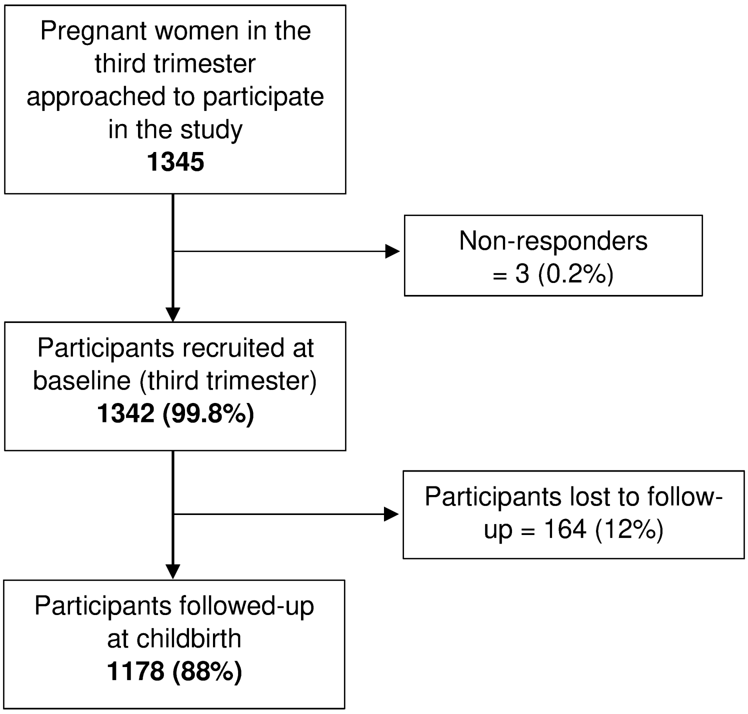

Figure S2: Predicted probability of PPH observed by fitting an interaction between Hb and D-dimer

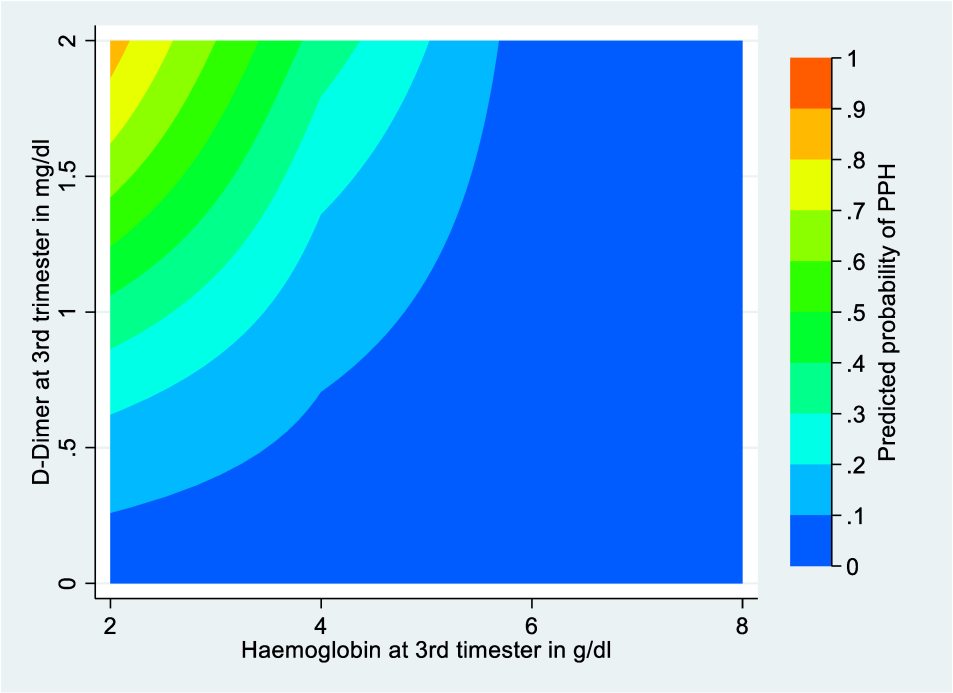

Supplement: Supplementary data [file bmjopen-2021-050815supp001.pdf]
