# Supplementary material for: Relationship between anaemia, coagulation parameters during pregnancy and postpartum haemorrhage at childbirth: a prospective cohort study
Source: BMJ Open. 2021 Oct 3;11(10):e050815. doi: 10.1136/bmjopen-2021-050815 (PMC8491293; doi:10.1136/bmjopen-2021-050815)
Supplement: Author's manuscript [file bmjopen-2021-050815.draft_revisions.pdf]

BMJ Open

BMJ Open is committed to open peer review. As part of this commitment we make the peer review history of every article we publish publicly available.

When an article is published we post the peer reviewers' comments and the authors' responses online. We also post the versions of the paper that were used during peer review. These are the versions that the peer review comments apply to.

The versions of the paper that follow are the versions that were submitted during the peer review process. They are not the versions of record or the final published versions. They should not be cited or distributed as the published version of this manuscript.

BMJ Open is an open access journal and the full, final, typeset and author-corrected version of record of the manuscript is available on our site with no access controls, subscription charges or pay-per-view fees (http://bmjopen.bmj.com).

If you have any questions on BMJ Open's open peer review process please email info.bmjopen@bmj.com

BMJ Open

Relationship between anaemia, coagulation parameters during pregnancy and postpartum haemorrhage at childbirth: a prospective cohort study

Journal:	*BMJ Open*
Manuscript ID	bmjopen-2021-050815
Article Type:	Original research
Date Submitted by the Author:	02-Mar-2021
Complete List of Authors:	Nair, Manisha; Oxford University, NPEU, Nuffield Department of Population Health<br>Chhabra, Shakuntala ; Mahatma Gandhi Institute of Medical Sciences<br>Choudhury , Saswati; Gauhati Medical College and Hospital<br>Deka, Dipika ; Srimanta Sankaradeva University of Health Sciences<br>Deka, Gitanjali ; Tezpur Medical College<br>Kakoty, Swapna ; Fakhruddin Ali Ahmed Medical College and Hospital<br>Kumar, Pramod ; Mahatma Gandhi Institute of Medical Sciences<br>Mahanta, Pranabika ; Jorhat Medical College and Hospital<br>Medhi, Robin ; Fakhruddin Ali Ahmed Medical College and Hospital<br>Rani, Anjali; Banaras Hindu University Institute of Medical Sciences, Department of Obstetrics and Gynaecology<br>Rao, Seeresha; Silchar Medical College and Hospital<br>Roy, Indrani ; Nazareth Hospital<br>Solomi V, Carolin ; Makunda Christian Leprosy and General Hospital<br>Talukdar, Ratna ; Jorhat Medical College and Hospital<br>Zahir, Farzana; Assam Medical College, Department of Obstetrics and Gynaecology<br>Kansal, Nimmi ; Dr Lal Pathlabs, National Reference Laboratory<br>Arora, Anil; Dr Lal PathLabs, National Reference Laboratory<br>Opondo, Charles; Oxford University, Nuffield Department of Population Health<br>Armitage, Jane; Oxford University, Nuffield Department of Population Health<br>Laffan, Michael; Imperial College London Faculty of Medicine, Haemostasis & Thrombosis<br>Stanworth, Simon; Oxford University, Department of Haematology/Transfusion Medicine<br>Quigley, Maria; Oxford University, National Perinatal Epidemiology Unit<br>Baigent, Colin; Oxford University, Nuffield Department of Population Health<br>Knight, Marian; Oxford University, National Perinatal Epidemiology Unit<br>Kurinczuk, Jennifer; Oxford University, National Perinatal Epidemiology Unit
Keywords:	Anaemia < HAEMATOLOGY, EPIDEMIOLOGY, OBSTETRICS

For peer review only - http://bmjopen.bmj.com/site/about/guidelines.xhtml

I, the Submitting Author has the right to grant and does grant on behalf of all authors of the Work (as defined in the below author licence), an exclusive licence and/or a non-exclusive licence for contributions from authors who are: i) UK Crown employees; ii) where BMJ has agreed a CC-BY licence shall apply, and/or iii) in accordance with the terms applicable for US Federal Government officers or employees acting as part of their official duties; on a worldwide, perpetual, irrevocable, royalty-free basis to BMJ Publishing Group Ltd ("BMJ") its licensees and where the relevant Journal is co-owned by BMJ to the co-owners of the Journal, to publish the Work in this journal and any other BMJ products and to exploit all rights, as set out in our licence.

The Submitting Author accepts and understands that any supply made under these terms is made by BMJ to the Submitting Author unless you are acting as an employee on behalf of your employer or a postgraduate student of an affiliated institution which is paying any applicable article publishing charge ("APC") for Open Access articles. Where the Submitting Author wishes to make the Work available on an Open Access basis (and intends to pay the relevant APC), the terms of reuse of such Open Access shall be governed by a Creative Commons licence – details of these licences and which Creative Commons licence will apply to this Work are set out in our licence referred to above.

Other than as permitted in any relevant BMJ Author's Self Archiving Policies, I confirm this Work has not been accepted for publication elsewhere, is not being considered for publication elsewhere and does not duplicate material already published. I confirm all authors consent to publication of this Work and authorise the granting of this licence.

For peer review only

Relationship between anaemia, coagulation parameters during pregnancy and postpartum haemorrhage at childbirth: a prospective cohort study

Authors

1. Manisha Nair, DPhil
National Perinatal Epidemiology Unit, Nuffield Department of Population Health,
University of Oxford, Old Road Campus, Headington, Oxford, OX3 7LF, UK
Email: manisha.nair@npeu.ox.ac.uk

2. Shakuntala Chhabra, MD
Mahatma Gandhi Institute of Medical Sciences,
Sevagram, Maharashtra 442102, India
Email: schhabra@mgims.ac.in

3. Saswati S Choudhury, MD
Gauhati Medical College and Hospital (GMCH)
Bhangagarh, Guwahati, Assam 781032, India
Email: saswatischoudhury@gmail.com

4. Dipika Deka, MD
Srimanta Sankaradeva University of Health Sciences,
Narkashur Hilltop, Bhangagarh, Guwahati, Assam 781032, India
Email: dpk.deka@gmail.com

5. Gitanjali Deka, MD
Tezpur Medical College
NH 15, Tezpur, Assam 784153, India
Email: gitanjali.dk@gmail.com

6. Swapna D Kakoty, MD
Fakhruddin Ali Ahmed Medical College and Hospital (FAAMCH)
Barpeta-Hospital-Jania Rd, Joti Gaon, Assam 781301, India
Email: swapna_kakoty@rediffmail.com

7. Pramod Kumar, MD
Mahatma Gandhi Institute of Medical Sciences,
Sevagram, Maharashtra 442102, India
Email: pkumar@mgims.ac.in

8. Pranabika Mahanta, MD
Jorhat Medical College and Hospital
Kushal Konwar Path, Barbheta, Jorhat, Assam 785001, India
Email: drpranabikamahanta@gmail.com

9. Robin Medhi, MD
Fakhruddin Ali Ahmed Medical College and Hospital (FAAMCH)
Barpeta-Hospital-Jania Rd, Joti Gaon, Assam 781301, India
Email: robinmedhi@yahoo.com

For peer review only

10. Anjali Rani, MD
Institute of Medical Sciences, Banaras Hindu University
Aurobindo Colony, Banaras Hindu University Campus, Varanasi, Uttar Pradesh 221005, India
Email: anjalirani.imsbhu@gmail.com

11. Seeresha Rao, MD
Silchar Medical College and Hospital (SMCH), Ghungoor Road, Masimpur, Uttar Krishnapur Pt III, Assam 788014, India
Email: sereesha2000@gmail.com

12. Indrani Roy, MD
Nazareth Hospital
Arbuthnot Rd, Nongkynrih, Laitumkhrah, Shillong, Meghalaya 793003, India
Email: indraniroydeb@gmail.com

13. Carolin Solomi V, MS
Makunda Christian Leprosy and General Hospital
Bazaricherra, Karimganj, Assam 788727, India
Email: dr_carolin@rediffmail.com

14. Ratna Kanta Talukdar, MD
Jorhat Medical College and Hospital
Kushal Konwar Path, Barbheta, Jorhat, Assam 785001, India
Email: rktalukdar@gmail.com

15. Farzana Zahir, MD
Assam Medical College (AMC)
Barbari, Dibrugarh, Assam 786002, India
Email: sajeedfarah@hotmail.com

16. Nimmi Kansal, MD
National Reference Laboratory, Dr Lal PathLabs
B7 Rd, Block E, Sector 18, Rohini, New Delhi, Delhi 110085, India
Email: nimmi.kansal@lalpathlabs.com

17. Anil Arora, MD
National Reference Laboratory, Dr Lal PathLabs
B7 Rd, Block E, Sector 18, Rohini, New Delhi, Delhi 110085, India
Email: Anil.Arora@lalpathlabs.com

18. Charles Opondo, PhD
National Perinatal Epidemiology Unit, Nuffield Department of Population Health, University of Oxford, Old Road Campus, Headington, Oxford, OX3 7LF, UK
Email: charles.opondo@npeu.ox.ac.uk

19. Jane Armitage, MD
Clinical Trial Service Unit, Nuffield Department of Population Health, University of Oxford, Old Road Campus, Headington, Oxford, OX3 7LF, UK

For peer review only

For peer review only - http://bmjopen.bmj.com/site/about/guidelines.xhtml

Email: jane.armitage@ctsu.ox.ac.uk

20. Michael A Laffan, FRCPath
Department of Haematology, Faculty of Medicine at Imperial College, Hammersmith Hospital, London, W12 0HS, UK
Email: m.laffan@imperial.ac.uk

21. Simon J Stanworth, FRCPath
Department of Haematology, NHS Blood and Transplant/Oxford University Hospitals NHS Foundation Trust, John Radcliffe Hospital; Radcliffe Department of Medicine, University of Oxford, Oxford, OX3 9DU, UK
Email: simon.stanworth@nhsbt.nhs.uk

22. Maria Quigley, MSc
National Perinatal Epidemiology Unit, Nuffield Department of Population Health, University of Oxford, Old Road Campus, Headington, Oxford, OX3 7LF, UK
Email: maria.quigley@npeu.ox.ac.uk

23. Colin Baigent, FMedSci
MRC Population Health Research Unit, Nuffield Department of Population Health, University of Oxford, Old Road Campus, Headington, Oxford, OX3 7LF, UK
Email: colin.baigent@ndph.ox.ac.uk

24. Marian Knight, FRCPE
National Perinatal Epidemiology Unit, Nuffield Department of Population Health, University of Oxford, Old Road Campus, Headington, Oxford, OX3 7LF, UK
Email: marian.knight@npeu.ox.ac.uk

25. Jenny J Kurinczuk, FFPH
National Perinatal Epidemiology Unit, Nuffield Department of Population Health, University of Oxford, Old Road Campus, Headington, Oxford, OX3 7LF, UK
Email: jenny.kurinczuk@npeu.ox.ac.uk

on behalf of the MaatHRI collaborators^

Authors 2-15 contributed equally

^Details about the MaatHRI collaboration can be found on https://www.npeu.ox.ac.uk/maathri

Corresponding author

Manisha Nair
National Perinatal Epidemiology Unit, Nuffield Department of Population Health, University of Oxford, Old Road Campus, Headington, Oxford, OX3 7LF, UK
Email: manisha.nair@npeu.ox.ac.uk; Phone: 01865 617820

For peer review only

For peer review only - http://bmjopen.bmj.com/site/about/guidelines.xhtml

Abstract

Objectives: To investigate the association between coagulation parameters and severity of anaemia (moderate anaemia: haemoglobin (Hb) 7-9.9g/dl and severe anaemia: Hb<7g/dl) during pregnancy and relate these to postpartum haemorrhage (PPH) at childbirth.

Design: A prospective cohort study of pregnant women recruited in the third trimester and followed-up after childbirth.

Setting: Ten hospitals across four states in India.

Participants: 1342 pregnant women

Intervention: Not applicable

Methods: Hb and coagulation parameters: fibrinogen, D-dimer, D-dimer/fibrinogen ratio, platelets, and INR (International normalised ratio) were measured at baseline. Participants were followed-up to measure blood loss within two-hours after childbirth and PPH was defined based on blood loss and clinical assessment. Associations between coagulation parameters, Hb, anaemia and PPH were examined using multivariable logistic regression models.

Outcomes measures: Adjusted Odds Ratio (aOR) with 95% confidence interval(CI).

Results: In women with severe anaemia during the third trimester, the D-dimer was 27% higher, mean fibrinogen 117mg/dl lower, D-dimer/fibrinogen ratio 69% higher, and INR 12% higher compared to women with no/mild anaemia. Mean platelets in severe anaemia was $37.8X10^9$/L lower compared with women with moderate anaemia. Similar relationships with smaller effect sizes were identified for women with moderate anaemia compared with women with no/mild anaemia. Low Hb and high INR at third trimester of pregnancy independently increased the odds of PPH at childbirth, but the other coagulation parameters were not found to be significantly associated with PPH.

Conclusion: Altered blood coagulation profile in pregnant women with severe anaemia could be a risk factor for PPH and requires further evaluation.

ARTICLE SUMMARY

Strengths and limitations of this study

- This is the first study to investigate the role of coagulation in relation to the increased risk of PPH in women with moderate/ severe anaemia.
- The large prospective cohort study substantially removed the potential for reverse causation when estimating the effect of the coagulation parameters on PPH.
- Another strength is reproducibility because we examined the relationship of Hb with five different parameters of coagulation and all suggested a similar effect.

For peer review only - http://bmjopen.bmj.com/site/about/guidelines.xhtml

- The follow-up rate was 88% and the mean Hb concentration at baseline (exposure of interest) for 12% participants who could not be followed-up was not different from the participants who were followed-up.

Key words: anaemia, coagulation parameters, pregnancy, postpartum haemorrhage, cohort study

Word count: 3955

For peer review only

INTRODUCTION

Moderate (Hb7-9.9g/dl[1]) and severe anaemia (Hb<7g/dl[1]) during pregnancy not only increase the risk of postpartum haemorrhage (PPH), but also increase the risk of dying from PPH by several fold[2-4]. The public health problem of anaemia during pregnancy is graded as moderate-severe in 183 countries across the world with about 529 million pregnant women with anaemia in 2011, globally[5] and an estimated 295,000 maternal deaths annually[6]. Explanations include low Hb associated with reduced oxygen availability resulting in reduced uterine contractility and early fatigue causing uterine atony and PPH[7]. However, changes in the coagulation profile in anaemic pregnant women may also predispose them to an increased risk of bleeding.

Pregnancy is a state of physiological hypercoagulability, with an increase in fibrinogen and decrease in fibrinolytic activity, with increasing gestational age[8 9]. An expansion in plasma volume results in a physiological decrease in platelets, haematocrit and Hb during pregnancy[8], although the prothrombin time remains largely stable[8 10 11]. While the haemostatic changes in normal pregnancy are well described, there have been few investigations of the relationship and potential clinical implications of coagulation abnormalities in association with severe anaemia in pregnant women. D-dimer was shown to be useful in risk stratification to rule out pulmonary embolism and to limit exposure of suspected pregnant women to imaging[12], but the role of D-dimer and other coagulation parameters in risk stratification of PPH is not clear.

The primary objective of this study was to investigate the association between blood coagulation parameters (fibrinogen, D-dimer, D-dimer/fibrinogen ratio, platelets and INR (International normalised ratio)) and severity of anaemia during the third trimester of pregnancy. The secondary objective was to examine the relationship between anaemia and coagulation parameters during the third trimester and PPH at childbirth.

METHODS

Study design

A hospital-based prospective cohort study undertaken through the Maternal and perinatal Health Research collaboration, India (MaatHRI)[13].

Study population

All pregnant women >28 weeks of gestation, ≥18 years, and planning a vaginal birth in 10 MaatHRI collaborating hospitals across four states in India (Assam, Meghalaya, Uttar Pradesh and Maharashtra) were approached to participate in the study. The response rate was 99.8% and 1342 eligible pregnant women who provided written informed consent were recruited between October 2018 and May 2019. The women were followed-up during labour and childbirth and up to 48 hours postpartum.

Baseline data

Information was collected from women during the baseline assessment about sociodemographic characteristics, previous and current pregnancy problems, medical co-morbidities, and other pregnancy characteristics. Blood samples were collected to measure Hb, haematocrit, fibrinogen, D-dimer, platelets, prothrombin time (from which INR was derived), and if present, cause of anaemia (inferred from measurement of serum ferritin and Hb electrophoresis). Using the WHO definition for anaemia in pregnancy[1], women with Hb ≥10g/dl were classified as no/mild anaemia, 7-9.9g/dl as moderate and <7g/dl as severe anaemia. We generated a D-dimer/fibrinogen ratio that was used in other studies[9 14 15]. Since

For peer review only - http://bmjopen.bmj.com/site/about/guidelines.xhtml

INR is not influenced by the pregnancy state[10 11], using the standard cut-off, we classified pregnant women into high (>1.1) and low INR (≤1.1) groups. Other coagulation parameters were analysed as continuous variables.

Laboratory methods

The MaatHRI platform has a laboratory infrastructure through a partnership with a private laboratory in India[13]. Blood collection, processing, storage and analysis were standardised. All blood samples were analysed at the national laboratory. The assay methods, traceability and performance characteristics for each test were agreed with experts from the University of Oxford's Wolfson laboratory and the Indian laboratory partner. Supplementary Table S1 shows the traceability and in Table S2 we present the assay methods and their performance characteristics.

The laboratory measured time in transit for each sample and their quality. Depending upon the remoteness of the hospital, the transit time ranged between 12 and 72 hours. Three types of samples were collected: EDTA whole blood for Hb, haematocrit, platelets and Hb electrophoresis, serum for ferritin, and citrated plasma for D-dimer, fibrinogen and INR. Citrated plasma samples were centrifuged at 3700 revolutions per minute (RPM) for 10 minutes using centrifuge machines of same make and model in all study hospitals, and aliquot was prepared from supernatant plasma, frozen immediately and shipped with dry ice. Samples that were inadequate, in terms of quantity, stability, temperature and other quality indicators, were discarded. A variable, 'hospital-code', was generated to account for transit time and other known and unknown potential biases related to sample quality in the statistical analysis.

Follow-up data

Similar to other studies[16 17], a calibrated blood collection drape was used to objectively measure the amount of blood loss within two hours after childbirth. The same make and model of drape (PPH alert bag) was used in all study hospitals. The drape was placed immediately after the birth of the baby (before removing the placenta) and blood loss was measured from the calibrated and colour coded markings on the drape While maximum blood loss is just before and after the removal of placenta and up to one hour after childbirth, the drape was left in situ up to two hours during the post-birth observation period in the labour room if the woman continued to bleed[17]. The drape could not be used for women who had a caesarean section, in which case estimates of blood loss were measured by the obstetrician from the suction bottle and soaked sponges. Only pregnant women with a planned vaginal birth were recruited in the study, thus the participants who had a caesarean section were women who had an emergency section after spontaneous rupture of membranes. As a result, the suction bottle contained very little liquor thereby making the blood loss estimates more accurate. The objective measurement methods were in line with the recommendations of the American College of Obstetricians and Gynecologists (ACOG)[18]. ACOG acknowledges the difficulty in accurately measuring blood loss after childbirth, but recommends use of calibrated drapes and hospital-based protocols for collecting and measuring blood loss after childbirth, which are more accurate than visual estimation[18].

PPH was defined based on measured blood loss within two hours after childbirth (≥500ml for women who had a vaginal birth and ≥1000ml for women who had a caesarean birth) and clinician diagnosed PPH requiring management. This was similar to the methods used to define PPH in other studies[16 17]. We also collected information about the mode of birth, maternal complications at birth, admission to intensive care unit and maternal death.

For peer review only - http://bmjopen.bmj.com/site/about/guidelines.xhtml

Sample size

A priori sample size calculations were done for two primary parameters: D-dimer and fibrinogen (see Table-S3). Sample sizes were calculated for a range of expected changes in the mean concentrations of the parameters (10%, 20% and 30%) between the no/mild anaemia and moderate/severe anaemia groups taking power (1-β)=90%, α=5% (two-tailed), and n1=n2. A sample size of 1028 had adequate power to detect a mean difference of 10% in the concentration of D-dimer and fibrinogen between the two study groups assuming a mean of 0.11 mg/dl (SD=0.573) for D-dimer[19] and 379 mg/dl (SD=0.78) for fibrinogen[20] in the baseline groups. This was inflated by 15% to account for potential loses which led to a total sample of 1209, rounded off to 1200 (n1=n2=600). However, we further increased the sample size during the study, finally recruiting 1342 pregnant women, to examine the difference in the concentration of coagulation parameters between three groups: no/mild anaemia, moderate and severe anaemia.

Statistical analysis

Descriptive statistics were used for all blood parameters, participant characteristics at baseline and PPH at childbirth. We calculated and compared the mean Hb across categories of gestational age, and mean gestational age across the categories of anaemia using t-test with Bartlett's statistics for equal variances. We examined the distribution of the continuous variables, and blood parameters that were not normally distributed: D-dimer, D-dimer/fibrinogen ratio and INR, were log transformed to create a normal distribution. These were used as outcome variables in the primary analysis and multivariable linear regression models were used to examine their individual association with Hb and anaemia after controlling for known confounding variables including gestational age, maternal age, pregnancy induced hypertension (PIH), pre-existing medical problems and hospital-code. We conducted tests for linear trend and used Chi-square tests to assess heterogeneity in odds ratios across categories of anaemia. We also examined the presence of any non-linear relationships between Hb and the coagulation parameters. The analysis was repeated using haematocrit instead of Hb as the exposure variable to test reproducibility of the results.

For the secondary objective, we analysed the association of PPH with Hb, anaemia and the coagulation parameters using multivariable logistic regression analysis controlling for potential confounders and exploring significant interactions. We found the variable 'hospital-code' to be strongly correlated with PIH and PPH. To improve model parsimony, 'hospital-code' was not included in the multivariable analysis. To understand whether the effect of Hb on PPH was moderated or mediated by each of the coagulation parameters, we tested for interaction and conducted mediation analysis, respectively. Likelihood-ratio test was used to examine statistically significant interactions at p<0.1 considering a lower power for the sub-group analysis. Mediation analysis was undertaken using the generalisation to the Baron-Kenny approach to mediation analysis.[21]

Missing data for the blood parameters were related to samples being discarded due to quality issues, but not with the level of the parameter itself or Hb. Therefore, data in the study were considered missing at random (MAR) and complete case analysis was used. All results were considered significant at a two-tailed p-value of <0.05. Analyses were undertaken using Stata version 16, Special Edition (StataCorp, College Station, Texas, USA).

For peer review only - http://bmjopen.bmj.com/site/about/guidelines.xhtml

Patient and public involvement

Patient and public were not involved in the design, conduct or reporting of the study.

Ethics approval and participant consent

The study was approved by the institutional review boards (IRB) of each coordinating Indian institution, namely: Srimanta Sankaradeva University of Health Sciences, Guwahati, Assam; Nazareth hospital, Shillong, Meghalaya; Emmanuel Hospital Association, New Delhi; Mahatma Gandhi Institute of Medical Sciences, Sevagram, Maharashtra; and the Institute of Medical Sciences, Banaras Hindu University, Varanasi, Uttar Pradesh. It also received approval from the Government of India's Health Ministry's Screening Committee, the Indian Council of Medical Research, New Delhi and by the Oxford Tropical Research Ethics Committee (OxTREC), University of Oxford, UK.
Written informed consent was taken from all participants.

RESULTS

The baseline characteristics of the study population are presented in Table-1. The mean Hb was 10.3g/dl and mean gestational age at recruitment was 35.3 weeks. Mean Hb was similar in the different periods of gestational age (p=0.275, see Table-1) and gestational age was also similar across the categories of anaemia (p=0.128, see Table-1). The most common anaemia was iron deficiency (microcytic-hypochromic anaemia 19.8% and about 28.5% had serum ferritin <15 µg/L), but 12.5% of women had macrocytic anaemia and 14% an HbE trait or disease. The mean haematocrit was 21% in women with severe anaemia compared with 30% in women with moderate anaemia and 37% in women with mild/no anaemia. About 17% of the study population reported a problem during the current pregnancy. A total of eight women reported an antepartum haemorrhage, of these four were in the category of no/mild anaemia, three in moderate and one in the severe anaemia group.

Table-1: Baseline characteristics of the study population

**Characteristics at baseline (Total participants at baseline = 1342)**	**Mean (SD)**
Maternal age (in years); N=1334	24.5 (4.2)
Gestational age at baseline recruitment (in weeks); N=1342	35.3 (3.7)
**Blood parameters at baseline (unit of measure)**	**Mean (SD)**
Hb (Hb in g/dl); N=1326	10.3 (1.9)
Hb in g/dl by categories of gestational age (p=0.275)	
28-32 weeks	10.0 (1.8)
33-36 weeks	10.5 (1.9)
≥37weeks	10.4 (1.9)
Platelets (X 10^9/L); N=1305	195.6 (73.7)
Fibrinogen (mg/dl); N=1270	410.9 (129.2)
	**Median (IQR)**
D-dimer (mg/dl); N=1264	0.08 (0.07)
D-dimer/fibrinogen ratio; N=1250	0.0002 (.0002)
International normalised ratio (INR); N=1243	0.96 (0.12)
	**No. of women (%)**
Anaemia	
No/mild (Hb ≥10g/dl)	790 (58.9)

For peer review only - http://bmjopen.bmj.com/site/about/guidelines.xhtml

Moderate (Hb 7-9.9g/dl)	465 (34.6)
Severe (Hb<7g/dl)	71 (5.3)
Missing	16 (1.2)
Mean gestational age by categories of anaemia (p=0.128)	**Mean (SD)**
No/mild anaemia	35.4 (3.7)
Moderate anaemia	35.1 (3.8)
Severe anaemia	35.0 (3.7)
INR	
≤1.1	1126 (83.9)
>1.1	117 (8.7)
Missing	99 (7.4)
HbE	
Normal	1141 (85.0)
Trait	129 (9.6)
Disease	56 (4.2)
Missing	16 (1.2)
Microcytic-hypochromic anaemia	
No	1010 (75.3)
Yes	266 (19.8)
Missing	66 (4.9)
Macrocytic anaemia	
No	1110 (82.7)
Yes	168 (12.5)
Missing	64 (4.8)
Serum Ferritin	
≥ 15 µg/L	927 (69.1)
< 15 µg/L	383 (28.5)
Missing	32 (2.4)
Other pregnancy characteristics at baseline	
Pregnancy induced hypertension	
No	1290 (96.1)
Yes	46 (3.4)
Missing	6 (0.5)
Pre-existing medical problems (other than haemoglobinopathies)	
No	1281 (95.5)
Yes	58 (4.3)
Missing	3 (0.2)

*Pre-existing medical problems (excluding haemoglobinopathies) included diabetes, essential hypertension, rheumatic heart disease, hypothyroidism, urinary tract infection, kidney stone, appendicitis, gall bladder problems, ovarian tumour, pulmonary tuberculosis and Hepatitis C infection.

Key follow-up data are presented in Table-2. There was a 12% loss to follow-up, but no difference in mean Hb during the third trimester between women who were followed-up (10g/dl) and those not followed-up (10g/dl). A flow chart showing the study population is provided in Figure-S1.

For peer review only - http://bmjopen.bmj.com/site/about/guidelines.xhtml

Table-2: Key data from the follow-up

Follow-up (Total participants at follow-up=1178, 12% loss to follow-up)	N=1178
**PPH**	**No. of women (%)**
No	1159 (98.4)
Yes	19 (1.6)
**Mode of delivery**	
Vaginal birth	853 (72.0)
Caesarean birth	332 (28.0)

Association of coagulation parameters with Hb and anaemia

The results of the linear regression analyses are presented in Table-3 and Figures 1-6. All coagulation parameters were significantly associated with Hb and anaemia during the third trimester. The relationships were linear (inverse linear associations), except for platelets that had a non-linear inverted J-shaped association with Hb (Figure-1).

Table-3: Association of coagulation parameters with Hb and anaemia at third trimester

	**Outcome variables**			
	**D-dimer (mg/dl)**	**D-dimer**		
**Independent variables**	Median (IQR)	Unadjusted Coefficient (95% CI)	Adjusted* Coefficient (95% CI)	P value - test for linear trend
Hb	-	0.96 (0.94 to 0.97)	0.96 (0.94 to 0.98)	<0.001
No/mild anaemia	0.07 (0.06)	1 (ref)§	1 (ref)§	0.003£
Moderate anaemia	0.08 (0.07)	1.11 (1.02 to 1.20)	1.08 (0.99 to 1.17)	
Severe anaemia	0.10 (0.09)	1.25 (1.04 to 1.50)	1.27 (1.07 to 1.50)	
	**Fibrinogen (mg/dl)**	**Fibrinogen**		
	Mean (SE)	Unadjusted Coefficient (95% CI)	Adjusted* Coefficient (95% CI)	P value - test for linear trend
Hb	-	14.68 (11.11 to 18.24)	15.58 (12.08 to 19.09)	<0.001
No/mild anaemia	431.1 (4.8)	0 (Ref)	0 (Ref)	<0.001£
Moderate anaemia	390.5 (5.7)	-40.5 (-55.3 to -25.7)	-39.2 (-53.7 to -24.9)	
Severe anaemia	319.9 (12.7)	-111.1 (-143.4 to -78.9)	-117.2 (-148.3 to -86.1)	

For peer review only - http://bmjopen.bmj.com/site/about/guidelines.xhtml

For peer review only

	D-dimer/ fibrinogen ratio	D-dimer/fibrinogen ratio		
	Median (IQR)	Unadjusted Coefficient (95% CI)	Adjusted* Coefficient (95% CI)	P value - test for linear trend
Hb	-	0.92 (0.90 to 0.95)	0.93 (0.91 to 0.95)	<0.001
No/mild anaemia	0.00017 (0.00017)	1 (ref)§	1 (ref)§	<0.001£
Moderate anaemia	0.00020 (0.00020)	1.21 (1.09 to 1.34)	1.17 (1.06 to 1.29)	
Severe anaemia	0.00027 (0.00049)	1.63 (1.30 to 2.06)	1.69 (1.36 to 2.09)	
	**INR**	**INR**		
	Median (IQR)	Unadjusted Coefficient (95% CI)	Adjusted* Coefficient (95% CI)	P value - test for linear trend
Hb	-	0.98 (0.97 to 0.99)	0.99 (0.98 to 0.99)	0.001
No/mild anaemia	0.94 (0.13)	1 (ref)§	1 (ref)§	0.007£
Moderate anaemia	0.96 (0.1)	1.03 (0.99 to 1.06)	1.02 (0.98 to 1.05)	
Severe anaemia	0.99 (0.13)	1.09 (1.02 to 1.17)	1.12 (1.04 to 1.19)	
	**Platelets ($x10^9$/L)**	**Platelets**		
	Mean (SE)	Unadjusted Coefficient (95% CI)	Adjusted* Coefficient (95% CI)	P value - test for linear trend
Hb	-	-3.79 (-5.83 to -1.74)	-4.57 (-6.64 to -2.49)	NA
No/mild anaemia	187.4 (2.5)	-26.04 (-34.40 to -17.68)	-26.09 (-34.51 to -17.67)	NA£
Moderate anaemia	213.5 (3.6)	0 (ref)	0 (ref)	
Severe anaemia	168.3 (9.4)	-45.21 (-63.91 to -26.51)	-37.78 (-56.47 to -19.09)	

*Adjusted for gestational age, maternal age, PIH, pre-existing medical problems and hospital-code; §Exponent of Log, hence reference is '1' (instead of zero); NA – not applicable; £P value - test for linear trend

After adjustment, the D-dimer concentration was 8% (95%CI -1 to +17%) higher in women with moderate anaemia and 27% (95% CI 7 to 50%) higher in severe anaemia compared with no/mild anaemia (p-value for linear trend=0.003). In women with moderate anaemia, the mean fibrinogen concentration was 39.2mg/dl (95% CI 24–53.7.9mg/dl) lower, and in severe anaemia 117.2mg/dl (95% CI 86.1–148.3mg/dl) lower than in women with no/mild anaemia (p-value for trend <0.001). Consequently, the D-dimer/fibrinogen ratio was 17% (95% CI 6–

For peer review only - http://bmjopen.bmj.com/site/about/guidelines.xhtml

29%) and 69% (95% CI 36–100%) higher respectively in women with moderate and severe anaemia compared with women with no/mild anaemia (p-value for trend<0.001).

Given the inverted J-shaped association between Hb and platelets, the moderate anaemia group was taken as the comparator. Compared to women with moderate anaemia, those with no/mild anaemia had a mean platelet concentration 26 x 10^9/L (95% CI 17.7 to 34.5 x 10^9/L) lower, and those with severe anaemia 38 x 10^9/L (19.1 to 56.5 x 10^9/L) lower. The INR was 2% (95% CI -2 to 5%) and 12% (95% CI 4 to 19%) higher in women with moderate and severe anaemia respectively compared with women with no/mild anaemia. The odds of having a high INR (>1.1) decreased by 19% per g/dl increase in Hb (adjusted OR (aOR) 0.81, 95% CI 0.73 to 0.91, p<0.001). The odds of having a high INR in women with moderate anaemia was not significantly different from women with no/mild anaemia (aOR 1.12, 95% CI 0.69 to 1.84, p=0.647), but women with severe anaemia had more than five-fold higher odds of having a high INR (aOR 5.10, 95% CI 2.31 to 11.29, p<0.001).

The tests for heterogeneity showed that all odds ratios were significantly different across the categories of anaemia. Figures 2 to 6 show the relationship between the coagulation parameters and the categories of anaemia. The findings did not change when stratified by types of anaemia, although the 95% CI widened due to the small numbers in each stratified category. Furthermore, repeating the analyses using haematocrit as the exposure variable did not change the results materially (Table-S4).

Association of PPH at childbirth with Hb and anaemia in the third trimester of pregnancy

After adjusting for known confounders, the odds of having a PPH at childbirth *increased* by 22% per g/dl *decrease* in Hb (aOR 0.78, 95% CI 0.63 to 0.98). The adjusted odds of having a PPH was nearly two-fold higher in women with moderate anaemia and more than five-fold higher in women with severe anaemia compared with women with mild/no anaemia. There was a significant linear trend of increasing adjusted odds of PPH with increasing severity of anaemia (p-value for linear trend 0.035) (Table-4).

Table-4: Association of PPH at childbirth with Hb and anaemia

Independent variables	Outcome: PPH at childbirth		
	Unadjusted OR (95% CI)	Adjusted* OR (95% CI)	P value – test for linear trend
Anaemia			
No/mild	1 (ref)	1 (ref)	0.035
Moderate	1.84 (0.68 to 4.93)	1.82 (0.66 to 5.01)	
Severe	4.17 (1.08 to 16.12)	5.11 (1.19 to 21.93)	

*Adjusted for gestational age, maternal age, PIH, pre-existing medical problems and mode of birth

Association of PPH at childbirth with coagulation parameters in the third trimester of pregnancy

After adjusting for confounders, the odds of having a PPH increased by more than five-fold in women who had an INR >1.1 during the third trimester of pregnancy (Table-5). The other

For peer review only - http://bmjopen.bmj.com/site/about/guidelines.xhtml

coagulation parameters, D-dimer, fibrinogen and platelets were not significantly associated with PPH at childbirth (Table-5). Mediation analysis showed no significant mediation of the effect of Hb on PPH via any coagulation parameter. There was a pattern of increasing predicted probability of PPH with a decrease in Hb and increase in D-dimer (Figure S2). Nevertheless we did not find evidence of statistical interaction between Hb and D-dimer in their association with PPH (p-value 0.529). We did not find any significant interaction between Hb and the other coagulation parameters.

Table-5: Association between PPH at childbirth and coagulation parameters in the third trimester of pregnancy

**Outcome- PPH at childbirth**	**Predictors - Coagulation parameters in the third trimester of pregnancy**	
	Unadjusted OR (95% CI)	Adjusted* OR (95% CI)
	D-dimer	
No	1 (ref)	1 (ref)
Yes	1.00 (0.79 to 1.26)	1.03 (0.80 to 1.32)
	Fibrinogen	
No	1 (ref)	1 (ref)
Yes	1.00 (0.99 to 1.006)	1.00 (0.99 to 1.005)
	Platelets	
No	1 (ref)	1 (ref)
Yes	0.99 (0.98 to 1.001)	0.99 (0.98 to 1.002)
	INR >1.1	
No	1 (ref)	1 (ref)
Yes	1.76 (0.39 to 7.78)	5.74 (1.09 to 30.19)

*Regression models adjusted for gestational age, maternal age, PIH, pre-existing medical problems and mode of delivery; D-dimer in mg/L FEU; Fibrinogen in mg/dl; platelets x10^9/L

DISCUSSION

The study showed that pregnant women with severe anaemia during the third trimester of pregnancy had a higher D-dimer, lower fibrinogen and therefore a higher D-dimer/fibrinogen ratio than those with mild or moderate anaemia as well as a higher INR, after controlling for known confounders. Similar associations were observed among women with moderate anaemia with levels intermediate between severe and mild anaemia. Having a lower Hb and high INR (>1.1) during the third trimester of pregnancy was independently associated with a higher odds of PPH, but we did not find any association between PPH and the other coagulation parameters.

Studies[2 22], including our previous study in India[3], have repeatedly shown that pregnant women with anaemia (particularly severe anaemia) are at a higher risk of PPH. It is also known that low fibrinogen, high INR, and high D-dimer or other measures of fibrinolysis are associated with an increased risk of PPH[8] but to what extent these changes are associated with anaemia has not previously been described. The association between platelets and PPH is not clear[8], nor whether it is the total concentration or functionality of platelets that matter[8].

For peer review only - http://bmjopen.bmj.com/site/about/guidelines.xhtml

This study identified a new potential role of an impaired coagulation profile in pregnant women with anaemia that could lead to PPH. These potential associations are hypothesis generating for further research, both to understand the direct causal effects and the mechanisms by which the coagulation changes might exert an impact on anaemic women at childbirth. The primary observation was lower fibrinogen level in women with moderate and severe anaemia in the study population. It is known that fibrinogen levels increase by more than 200% during pregnancy[8] compared to the non-pregnant state to prevent haemorrhage during childbirth, and a recent meta-analysis of concentration of coagulation parameters by gestational age in pregnancy estimated a mean fibrinogen level of 556 (466-664) mg/dl during the third trimester of pregnancy[23]. Compared with this, mean fibrinogen levels during the third trimester were 391(379-402) mg/dl and 320(295-345) mg/dl in pregnant women with moderate and severe anaemia, respectively in the study population with a linear decrease in fibrinogen level by severity of anaemia, thus potentially increasing the risk of PPH.

There is some evidence that haemodilution has a profibrinolytic effect[24 25], thus another possibility is the presence of low grade pre-delivery fibrinolysis in pregnant women with severe anaemia in the study, which might also predispose them to higher blood loss or haemorrhage at childbirth. The median D-dimer levels in the study population in different categories of anaemia (Table-3) was comparable with the estimated mean D-Dimer during the third trimester of pregnancy in the meta-analysis[23], but we found a linear increase in D-dimer-to-fibrinogen ratio with increase in severity of anaemia. Under a conventional state of hypercoagulability during pregnancy, the decrease in fibrinogen should have been matched with a decrease in fibrinolytic activity, but in our study population with moderate and severe anaemia, the two processes seem to be operating in opposite directions, thereby creating a potential imbalance in clot formation and lysis which could increase the risk of PPH. Further, we also observed a pattern of low Hb and high D-dimer having a multiplicative effect on increased probability of PPH, although the interaction was not statistically significant. We did not find any underlying cause of blood loss (example placenta praevia or abruption), or antepartum haemorrhage in pregnant women with anaemia that could explain both low Hb and high D-dimer.

Likewise, the relative increase in INR in pregnant women with severe anaemia cannot be explained by the physiological changes in pregnancy as INR generally remains stable in pregnancy[11]. Women with severe anaemia had a low haematocrit (21%). While high haematocrit (>50%) is thought to artificially prolong PT from which INR is calculated, a low haematocrit (<25%) should not affect the measurement of PT using standard sodium citrate tubes[26]. It is possible that women with severe anaemia, who were mostly iron deficient, also have vitamin K deficiency due to malnutrition leading to an increase in INR. Prolongation of PT and increase in INR have been shown in patients with sickle cell disease, the increase being proportional to the severity of anaemia[27], and in a study of patients with haematological malignancies who were treated with chemotherapy[28], suggesting a delay in the initiation of the coagulation cascade in people with low Hb. This could explain the observed higher odds of PPH associated with high INR >1.1 in our study population.

We found an inverted J-shaped association between platelets and severity of anaemia. While the lower mean concentration of platelets in women with severe anaemia is in line with the impairment in the other coagulation parameters, the reasons for the lower mean concentration in no/mild anaemia compared with moderate anaemia is unclear. One possible explanation

For peer review only - http://bmjopen.bmj.com/site/about/guidelines.xhtml

could be residual confounding by pregnancy induced hypertension (PIH). Women who have severe PIH [e.g. HELLP (haemolysis, elevated liver enzymes, and low platelet count) syndrome] have low haemodilution (high Hb) and low platelets[29]. The relationship between anaemia and platelets is also unclear. *In vitro* studies show agglutination of platelets with lowering of Hb[30 31], others found an association between iron deficiency anaemia and thrombocytosis[32 33], and yet others suggest that anaemia impairs the role of red blood cells that normally push the platelets towards the vessel wall during the coagulation process to initiate clot formation[28 34].

Strengths and limitations

The main strength of this study is that it was large and prospective allowing examination of the relationship between Hb, anaemia and coagulation parameters during late pregnancy and their subsequent effects on blood loss at childbirth. Robust and standardised methods were employed to minimise bias, and improve the validity and reliability of the findings. The design allowed adjustment for gestational age, a major factor influencing coagulation parameters. The blood parameters were measured prospectively in the same laboratory in the third trimester of pregnancy (baseline) prior to labour and birth, and blood loss was measured at childbirth, addressing the risk of reverse causality. Another strength is reproducibility. We examined the relationship of Hb with five different parameters of coagulation and all suggested the same effect. We were also able to replicate the findings using haematocrit as the exposure variable.

The findings are generalizable to the population in India as data was collected from 10 hospitals across four states in India, which are different in terms of their socioeconomic contexts, healthcare facilities, food habits, prevalence of malnutrition and anaemia among pregnant women, and burden of maternal complications and death. The physiological changes associated with anaemia observed in our study are likely to be generalizable to all pregnant women, globally.

One limitation was the 12% loss to follow-up due to staff problems in one hospital. None of the participants in that hospital were followed up during or after childbirth, thus any bias due to loss to follow-up is likely to be minimal, as it was not related to the exposures or outcomes examined in the study. The mean Hb in women who were followed-up was not different from those who were not followed-up. Although we objectively measured blood loss at childbirth using a calibrated blood collection drape (for vaginal birth) and from suction bottle and soaked sponges (for caesarean birth), we cannot rule out measurement errors, but as mentioned earlier, the methods conformed to the recommendations of ACOG. In addition, there is no evidence that clinician estimated blood loss or blood loss measured by calibrated drape is associated with differential misclassification of PPH. Therefore, it is less likely that the results are influenced by the methods used for ascertaining PPH at childbirth. 1.6% of the study population had PPH which was comparable with the rate estimated in a previous study (1.1%)[35], but the lower number of events reduced the statistical power of the analysis for the secondary objective. Low number of events also limited the statistical power of the effect of the interaction between low Hb and high D-dimer on increased probability of PPH. Further, despite using standardised laboratory procedures and accounting for time taken for the blood samples to reach the national reference laboratory from the study hospitals, we cannot completely rule out measurement errors for the blood parameters.

For peer review only - http://bmjopen.bmj.com/site/about/guidelines.xhtml

Conclusion

In this study of pregnant women, measures of the coagulation parameters in the third trimester were significantly associated with the severity of anaemia. We identified a substantial independent effect of high INR and low Hb on increased risk of PPH at childbirth. Given the high prevalence of anaemia in pregnant women, globally, further studies are required to investigate the mechanisms through which coagulation parameters could increase the risk of PPH in pregnant women with anaemia.

Conflicts of interest: The authors declare that they have no competing interests.

Author statement: MN developed the concept and designed the study, conducted the statistical analysis, led the overall work as chief investigator, and wrote the first draft of the paper. SC, SSC, DD, GD, SDK, PK, PM, RM, AR, SR, IR, CSV, RKT, and FZ contributed equally, and their names are included in the alphabetic order of their last name. They are collaborators and investigators for the study, contributed to developing the study, and led the work in their respective institution. They also edited the paper. NK and AA contributed to developing the laboratory measures for the study, and AA edited the laboratory measurement section of the paper. CO provided statistical expertise, and contributed to writing the statistical methods and results. JA contributed to developing the concept for the study and edited the paper. SJS and MAL contributed to interpreting the results of the study and edited the paper. MQ provided statistical advice. CB, MK, JJK are advisors and have contributed to developing the study. JJK also edited the paper.

Ethics approval: The study was approved by the institutional review boards (IRB) of each coordinating Indian institution, namely: Srimanta Sankaradeva University of Health Sciences, Guwahati, Assam; Nazareth hospital, Shillong, Meghalaya; Emmanuel Hospital Association, New Delhi; Mahatma Gandhi Institute of Medical Sciences, Sevagram, Maharashtra; and the Institute of Medical Sciences, Banaras Hindu University, Varanasi, Uttar Pradesh. It also received approval from the Government of India's Health Ministry's Screening Committee, the Indian Council of Medical Research, New Delhi and by the Oxford Tropical Research Ethics Committee (OxTREC), University of Oxford, UK.

Funding: The study was funded by a Nuffield Department of Population Health (NDPH) Pump-priming award, and the MaatHRI platform is funded by a Medical Research Council Career Development Award to Manisha Nair (Grant Ref: MR/P022030/1). The funders had no role in the study design, data collection, analysis or writing of the report. Manisha Nair had full access to all the information for the paper and had final responsibility for the decision to submit for publication.

Data statement: The data and metadata used in this study are available for free and can be obtained by contacting the corresponding author.

Figure legends

Figure-1: Inverted J-shaped association between Hb and platelets

Figure-2: Relative difference in D-dimer across the categories of anaemia

Figure-3: Absolute difference in fibrinogen across the categories of anaemia

For peer review only - http://bmjopen.bmj.com/site/about/guidelines.xhtml

Figure-4: Relative difference in D-dimer/fibrinogen ratio across the categories of anaemia

Figure-5: Absolute difference in platelets across the categories of anaemia

Figure-6: Relative difference in INR across the categories of anaemia

Supporting information

Table-S1: Traceability of Assays

Table-S2: Assay Information and performance characteristics

Table-S3: Sample size calculations

Table-S4: Association of coagulation parameters with haematocrit at third trimester

Figure S1: Flow chart showing the study population

Figure S2: Predicted probability of PPH observed by fitting an interaction between Hb and D-dimer

REFERENCES

1. World Health Organisation. Nutritional anaemias: tools for effective prevention and control. Geneva: WHO, 2017.

2. Kavle JA, Stoltzfus RJ, Witter F, et al. Association between anaemia during pregnancy and blood loss at and after delivery among women with vaginal births in Pemba Island, Zanzibar, Tanzania. *Journal of health, population, and nutrition* 2008;26(2):232-40. [published Online First: 2008/08/09]

3. Nair M, Choudhury MK, Choudhury SS, et al. Association between maternal anaemia and pregnancy outcomes: a cohort study in Assam, India. *BMJ global health* 2016;1(1):e000026. doi: 10.1136/bmjgh-2015-000026 [published Online First: 2016/04/07]

4. Guignard J, Deneux-Tharaux C, Seco A, et al. Gestational anaemia and severe acute maternal morbidity: a population-based study. *Anaesthesia* 2020;n/a(n/a) doi: 10.1111/anae.15222

5. World Health Organisation. The global prevalence of anaemia in 2011. Geneva: WHO, 2015.

6. Trends in maternal mortality: 2000 to 2017: estimates by WHO, UNICEF, UNFPA, World Bank Group and the United Nations Population Division. Geneva: World Health Organization, 2019.

7. Pavord S, Daru J, Prasannan N, et al. UK guidelines on the management of iron deficiency in pregnancy. *British Journal of Haematology* 2020;188(6):819-30. doi: 10.1111/bjh.16221

8. Thornton P, Douglas J. Coagulation in pregnancy. *Best practice & research Clinical obstetrics & gynaecology* 2010;24(3):339-52. doi: 10.1016/j.bpobgyn.2009.11.010 [published Online First: 2010/01/26]

9. Réger B, Péterfalvi Á, Litter I, et al. Challenges in the evaluation of D-dimer and fibrinogen levels in pregnant women. *Thrombosis research* 2013;131(4):e183-e87. doi: https://doi.org/10.1016/j.thromres.2013.02.005

10. Katz D, Beilin Y. Disorders of coagulation in pregnancy. *BJA: British Journal of Anaesthesia* 2015;115(suppl_2):ii75-ii88. doi: 10.1093/bja/aev374

11. Szecsi PB, Jorgensen M, Klajnbard A, et al. Haemostatic reference intervals in pregnancy. *Thrombosis and haemostasis* 2010;103(4):718-27. doi: 10.1160/th09-10-0704 [published Online First: 2010/02/23]

For peer review only - http://bmjopen.bmj.com/site/about/guidelines.xhtml

12. van der Pol LM, Tromeur C, Bistervels IM, et al. Pregnancy-Adapted YEARS Algorithm for Diagnosis of Suspected Pulmonary Embolism. *New England Journal of Medicine* 2019;380(12):1139-49. doi: 10.1056/NEJMoa1813865
13. Nair M, Bezbaruah B, Bora A, et al. Maternal and perinatal Health Research Collaboration, India (MaatHRI): methodology for establishing a hospital-based research platform in a low and middle income country setting. *F1000Research* 2020
14. Hajsadeghi S, Kerman SR, Khojandi M, et al. Accuracy of D-dimer:fibrinogen ratio to diagnose pulmonary thromboembolism in patients admitted to intensive care units. *Cardiovasc J Afr* 2012;23(8):446-56. doi: 10.5830/CVJA-2012-041
15. Kara H, Bayir A, Degirmenci S, et al. D-dimer and D-dimer/fibrinogen ratio in predicting pulmonary embolism in patients evaluated in a hospital emergency department. *Acta clinica Belgica* 2014;69(4):240-5. doi: 10.1179/2295333714y.0000000029 [published Online First: 2014/07/12]
16. Sentilhes L, Winer N, Azria E, et al. Tranexamic Acid for the Prevention of Blood Loss after Vaginal Delivery. 2018;379(8):731-42. doi: 10.1056/NEJMoa1800942
17. Widmer M, Piaggio G, Nguyen TMH, et al. Heat-Stable Carbetocin versus Oxytocin to Prevent Hemorrhage after Vaginal Birth. 2018;379(8):743-52. doi: 10.1056/NEJMoa1805489
18. American College of Obstetricians and Gynecologists. Quantitative Blood Loss in Obstetric Hemorrhage. Washington, DC: ACOG, 2019.
19. Kline JA, Williams GW, Hernandez-Nino J. D-dimer concentrations in normal pregnancy: new diagnostic thresholds are needed. *Clinical chemistry* 2005;51(5):825-9. doi: 10.1373/clinchem.2004.044883 [published Online First: 2005/03/15]
20. Adler G, Duchinski T, Jasinska A, et al. Fibrinogen Fractions in the Third Trimester of Pregnancy and in Puerperium. *Thrombosis Research* 2000;97(6):405-10. doi: http://dx.doi.org/10.1016/S0049-3848(99)00190-5
21. Imai K, Keele L, Tingley D. A general approach to causal mediation analysis. *Psychological methods* 2010;15(4):309-34. doi: 10.1037/a0020761 [published Online First: 2010/10/20]
22. Malhotra M, Sharma JB, Batra S, et al. Maternal and perinatal outcome in varying degrees of anemia. *International journal of gynaecology and obstetrics: the official organ of the International Federation of Gynaecology and Obstetrics* 2002;79(2):93-100. doi: 10.1016/s0020-7292(02)00225-4 [published Online First: 2002/11/13]
23. Tang J, Lin Y, Mai H, et al. Meta-analysis of reference values of haemostatic markers during pregnancy and childbirth. *Taiwanese Journal of Obstetrics and Gynecology* 2019;58(1):29-35. doi: https://doi.org/10.1016/j.tjog.2018.11.004
24. Bolliger D, Szlam F, Levy JH, et al. Haemodilution-induced profibrinolytic state is mitigated by fresh-frozen plasma: implications for early haemostatic intervention in massive haemorrhage. *British journal of anaesthesia* 2010;104(3):318-25. doi: 10.1093/bja/aeq001 [published Online First: 2010/02/06]
25. Bolliger D, Szlam F, Molinaro RJ, et al. Finding the optimal concentration range for fibrinogen replacement after severe haemodilution: an in vitro model. *British journal of anaesthesia* 2009;102(6):793-9. doi: 10.1093/bja/aep098 [published Online First: 2009/05/08]
26. Siegel JE, Swami VK, Glenn P, et al. Effect (or lack of it) of severe anemia on PT and APTT results. *American journal of clinical pathology* 1998;110(1):106-10. doi: 10.1093/ajcp/110.1.106 [published Online First: 1998/07/14]
27. Antwi-Baffour S, Kyeremeh R, Annison L. Severity of Anaemia Has Corresponding Effects on Coagulation Parameters of Sickle Cell Disease Patients. *Diseases* 2019;7(4):59. doi: 10.3390/diseases7040059
28. Roeloffzen WWH, Kluin-Nelemans HC, Bosman L, et al. Effects of red blood cells on hemostasis. *Transfusion* 2010;50(7):1536-44. doi: 10.1111/j.1537-2995.2010.02586.x

For peer review only

29. Abildgaard U, Heimdal K. Pathogenesis of the syndrome of hemolysis, elevated liver enzymes, and low platelet count (HELLP): a review. *European Journal of Obstetrics & Gynecology and Reproductive Biology* 2013;166(2):117-23. doi: https://doi.org/10.1016/j.ejogrb.2012.09.026
30. Scharbert G, Wetzel L, Berlinger L, et al. Effect of anemia on coagulation and platelet function: a whole blood in vitro study. *Crit Care* 2011;15(Suppl 1):P445-P45. doi: 10.1186/cc9865 [published Online First: 2011/03/11]
31. Horne MK, 3rd, Cullinane AM, Merryman PK, et al. The effect of red blood cells on thrombin generation. *Br J Haematol* 2006;133(4):403-8. doi: 10.1111/j.1365-2141.2006.06047.x [published Online First: 2006/04/29]
32. Kadikoylu G, Yavasoglu I, Bolaman Z, et al. Platelet parameters in women with iron deficiency anemia. *J Natl Med Assoc* 2006;98(3):398-402.
33. Soto AF, Ford P, Mastoris J. Thrombocytosis in Iron Deficiency Anemia: What the Primary Care Physician Needs To Know. *Blood* 2006;108(11):3723-23. doi: 10.1182/blood.V108.11.3723.3723
34. Uijttewaal WS, Nijhof EJ, Bronkhorst PJ, et al. Near-wall excess of platelets induced by lateral migration of erythrocytes in flowing blood. *The American journal of physiology* 1993;264(4 Pt 2):H1239-44. doi: 10.1152/ajpheart.1993.264.4.H1239 [published Online First: 1993/04/01]
35. Nair M, Choudhury MK, Choudhury SS, et al. IndOSS-Assam: investigating the feasibility of introducing a simple maternal morbidity surveillance and research system in Assam, India. 2016;1(1):e000024. doi: 10.1136/bmjgh-2015-000024 %J BMJ Global Health

For peer review only

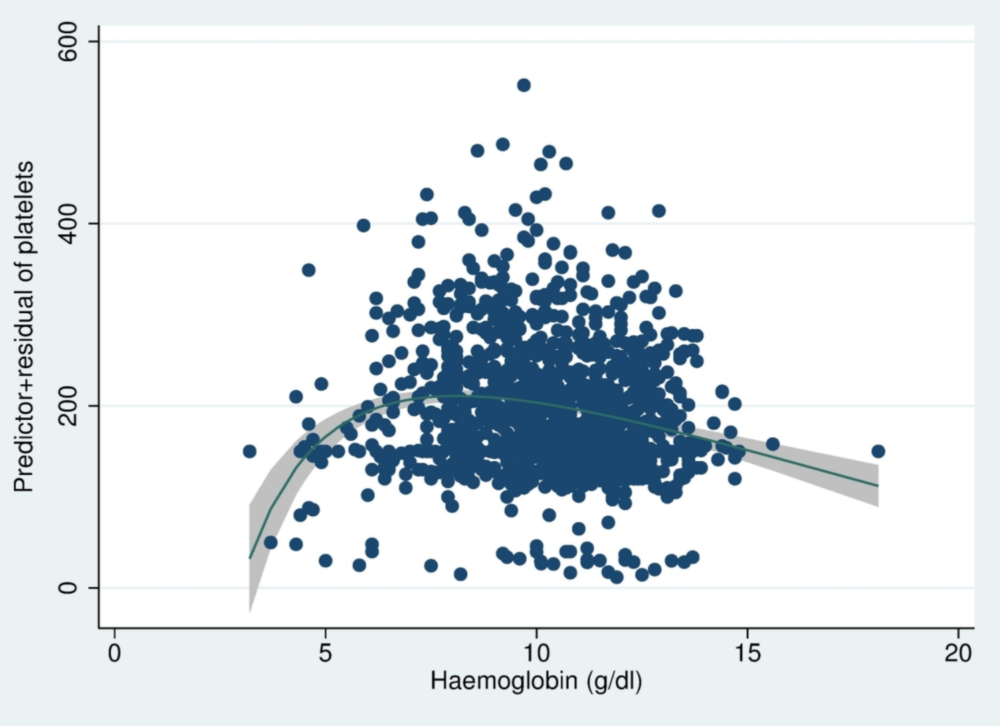

Figure-1: Inverted J-shaped association between Hb and platelets

139x101mm (300 x 300 DPI)

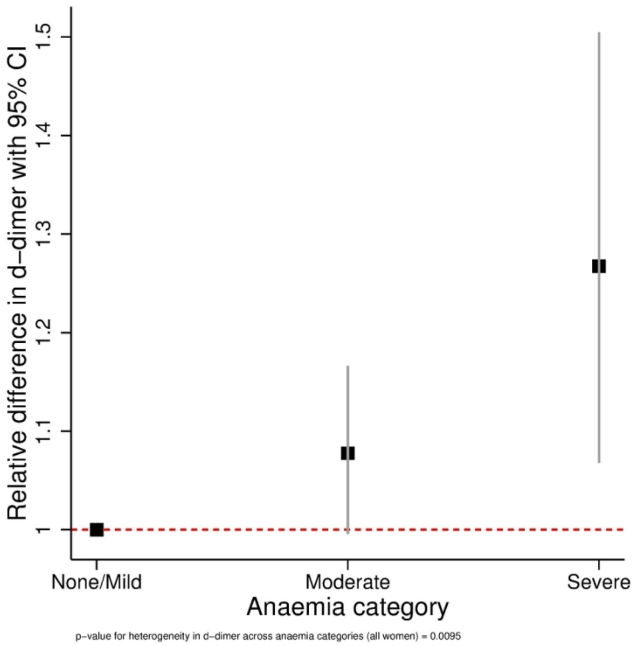

Figure-2: Relative difference in D-dimer across the categories of anaemia

139x101mm (300 x 300 DPI)

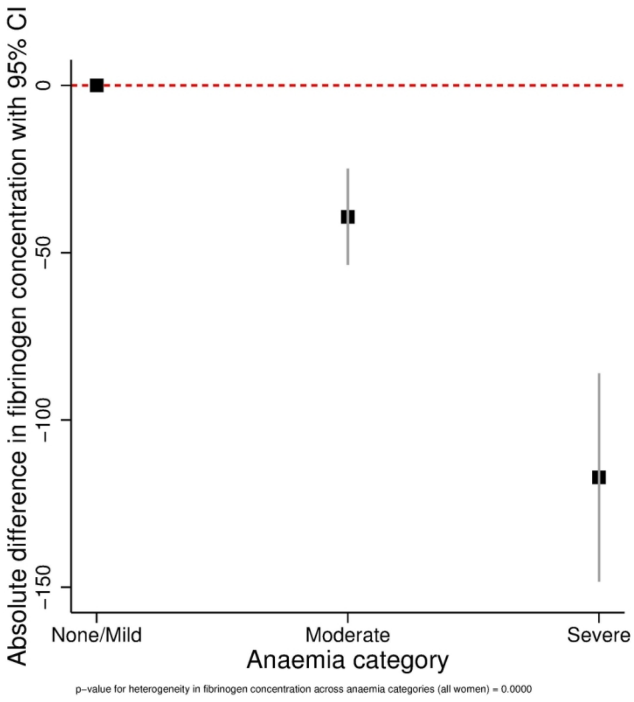

Figure-3: Absolute difference in fibrinogen across the categories of anaemia

139x101mm (300 x 300 DPI)

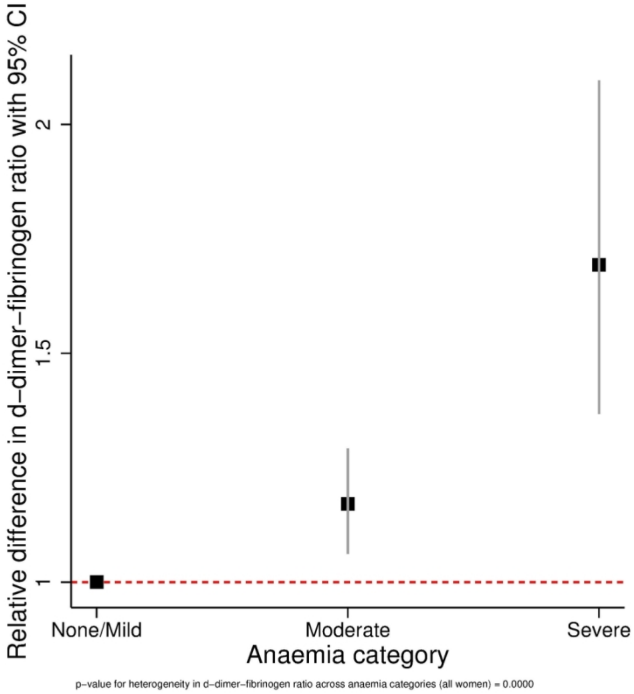

Figure-4: Relative difference in D-dimer/fibrinogen ratio across the categories of anaemia

139x101mm (300 x 300 DPI)

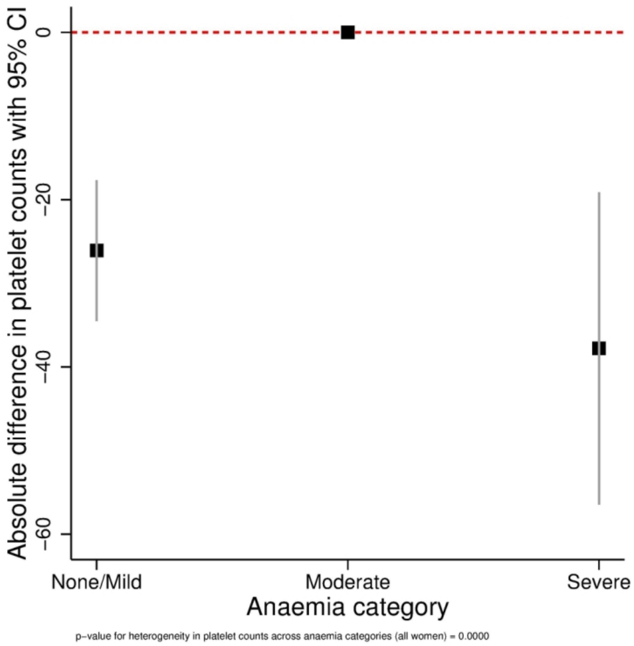

Figure-5: Absolute difference in platelets across the categories of anaemia

139x101mm (300 x 300 DPI)

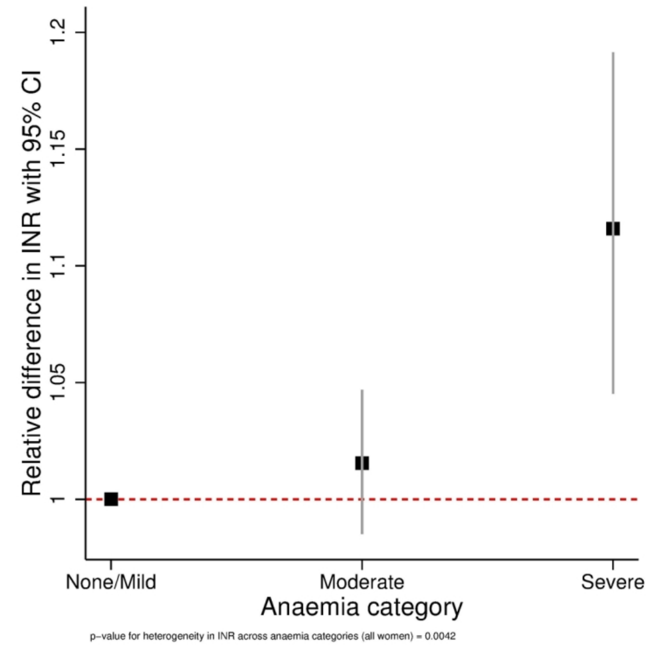

Figure-6: Relative difference in INR across the categories of anaemia

139x101mm (300 x 300 DPI)

Table-S1: Traceability of Assays

Sl No	Name of test	Calibrator traceability (reference material/ reference method)	Units	Typical Calibrator value	Calibrator uncertainty of measurement
1	Haemoglobin	1:250 dilution in NCCLS2 recommended reagent for the hemiglobincyanide (cyanmethemoglobin	g/dl	12.58	1.00%
2	Hematocrit	Calculated	%	calculated	NA
3	Platelets	A 1:101 dilution is made using a 20 µL TC pipette and 2 mL of 1% filtered ammonium oxalate (CLSI/ formerly NCCLS)	thou/mm3	214.1	6.00%
4	Serum Ferritin	WHO 3rd International Standard 94/572	ng/ml	Low 5.44<br>High 953	Low 19.5<br>High 9.3
5	Haemoglobin electrophoresis	NGSP Certification for A2/F	%	HbF-6.6 % and HbA2-6.7 %	HbF- Low- NA<br>High 1.8 %<br>HbA2- Low-NA, High- 3.6 %
6	D-Dimer	Pre-calibrated	mg/L FEU	NA	NA
7	Fibrinogen, Clotting activity	WHO	mg/dl	269	2.7%
8	International Normalized Ratio (INR)	Calculated			

NGSP - National Glycohemoglobin Standardization Program; CLSI – Clinical and Laboratory Standards Institute; HbF – Fetal haemoglobin; HbA2 - Haemoglobin Subunit Alpha 2; NA - Not applicable; WHO – World Health Organisation

For peer review only

Table-S2: Assay Information and performance characteristics

Sl No	Name of test	System used for the analysis	Method information (supplier/ method)	Manufacturers' Analytical Range	Laboratory Reportable range	Normal Reference range	Biological variation	Uncertainty of measurement	QC material	External Quality Assurance
1	Haemoglobin	DxH -800 (Beckman Coulter, Fullerton, CA, USA)	Photometric	0.0-99.9g/dl	1.25	13.-17.0g/dl (adult male)	2.1	4.0	Coulter 6c cell control	CAP
2	Hematocrit	DxH -800 (Beckman Coulter, Fullerton, CA, USA)	Automated calculation	0.99.9	NA	40-50% (adult male)	1.9	4.0	Coulter 6c cell control	CAP
3	Platelets	DxH -800 (Beckman Coulter, Fullerton, CA, USA)	Impedance/ Coulter principle	0.7000	10-1000	150-450thou/mm3 (adult male)	2.9	6.0	Coulter 6c cell control	CAP
4	Serum Ferritin	Siemens ADVIA Centaur	Chemiluminescence Immunoassay (CLIA)	0.5 – 1650 ng/ml	<0.5, >16500		14.2	22.5	BIO-RAD	CAP PT
5	Haemoglobin electrophoresis	Variant II Hemoglobin testing system (Bio-Rad, Hercules, CA, USA)	High Performance Liquid Chromatography	HbF-1.3-44.3 % HbA2-1.6-18.7 %	HbF-1.3-99.8% HbA2-1.6-18.7 %	HbF- <1.5 % HbA2-1.5-3.5 %	HbF-5.6 % HbA2-4.4 %	HbF-10.97 % HbA2-8.62 %	BIO-RAD	CAP
6	D-Dimer	STA-R Evolution (Diagnostica Stago, Cedex, France)	Latex-enhanced immunoturbidimetry	0.22-20.0	0.22-20.0	< 0.50	10.4	20.38	Stago	CAP
7	Fibrinogen, Clotting activity	Sysmex CS5100 analyzer (Sysmex Corporation, Kobe, Japan)	Photo optical clot detection	30 -1400	50 -1200	200 - 400	13.8	27.05	Siemens	CAP
8	International Normalized Ratio (INR)	Calculated from Prothrombin time measured by Photo-optical clot Detection on Sysmex CS5100 analyzer (Sysmex Corporation, Kobe, Japan)	Calculated	Calculated	NA	0.9 – 1.1	NA	NA	Siemens	CAP

INR: International Normalized Ratio; NABL: National Accreditation Board for Testing and Calibration; CAP: College of American Pathologists; CAP PT - College of American Pathologists Proficiency Testing programme; HbF: Fetal haemoglobin; HbA2: Haemoglobin Subunit Alpha; QC: Quality Control; NA - Not applicable

For peer review only - http://bmjopen.bmj.com/site/about/guidelines.xhtml

Table-S3: Sample size calculations

Coagulation parameter	Concentration in general pregnant population Mean (SD)	Sample required for a range of expected change in concentration of the blood parameters in either direction					
		10% change		20% change		30% change	
		n1	Total	n1	Total	n1	Total
D-Dimer (mg/dl)	0.11 (0.573)[7]	**514**	**1028**	129	258	58	116
Fibrinogen (mg/dl)	379 (0.78)[8]	90	180	23	46	10	20

n1 – women with haemoglobin concentration <10g/dl; n2 – women with haemoglobin concentration ≥10g/dl

Table-S4: Association of coagulation parameters with haematocrit at third trimester

Independent variables	Outcome variables	
	D-Dimer	
	Unadjusted OR (95% CI)	Adjusted* OR (95% CI)
Haematocrit (HCT)	0.99 (0.98 to 0.99)	0.99 (0.98 to 0.99)
HCT ≥30%	1 (ref)	1 (ref)
HCT <30%	1.13 (1.03 to 1.25)	1.13 (1.03 to 1.24)
	Fibrinogen	
	Unadjusted OR (95% CI)	Adjusted* OR (95% CI)
Haematocrit (HCT)	4.57 (3.37 to 5.77)	5.69 (4.51 to 6.88)
HCT ≥30%	0 (Ref)	0 (Ref)
HCT <30%	-58.3 (-75.4 to -41.1)	-68.1 (-84.8 to -51.3)
	D-Dimer/Fibrinogen ratio	
	Unadjusted OR (95% CI)	Adjusted* OR (95% CI)
Haematocrit (HCT)	0.98 (0.97 to 0.99)	0.97 (0.96 to 0.98)
HCT ≥30%	1 (ref)	1 (ref)
HCT <30%	1.35 (1.19 to 1.52)	1.39 (1.24 to 1.56)
	INR	
	Unadjusted OR (95% CI)	Adjusted* OR (95% CI)
Haematocrit (HCT)	0.99 (0.99 to 1.00)	0.99 (0.99 to 1.00)
HCT ≥30%	1 (ref)	1 (ref)
HCT <30%	1.02 (0.98 to 1.06)	1.05 (1.01 to 1.09)
	Platelets	
	Unadjusted OR (95% CI)	Adjusted* OR (95% CI)
Haematocrit (HCT)	-0.38 (-1.07 to 0.30)	-0.89 (-1.59 to -0.18)
HCT ≥30%	0 (ref)	0 (ref)
HCT <30%	4.84 (-4.93 to 14.61)	11.32 (1.42 to 21.23)

*Adjusted for gestational age, maternal age, PIH, pre-existing medical problems and hospital-code.

For peer review only

For peer review only - http://bmjopen.bmj.com/site/about/guidelines.xhtml

Figure-S1: Flow chart showing the study population

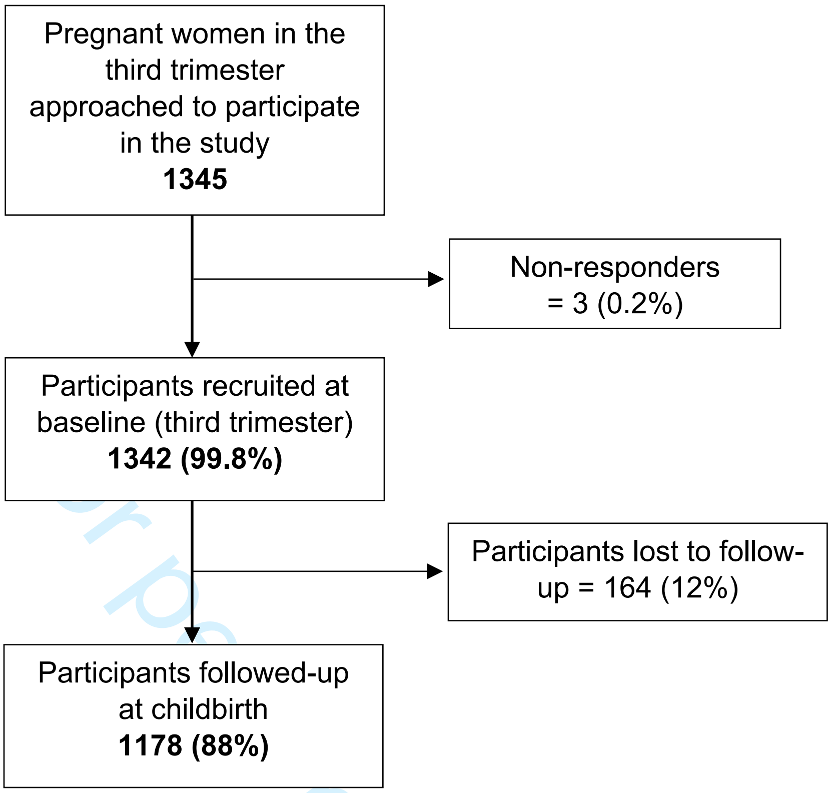

Figure S2: Predicted probability of PPH observed by fitting an interaction between Hb and D-dimer

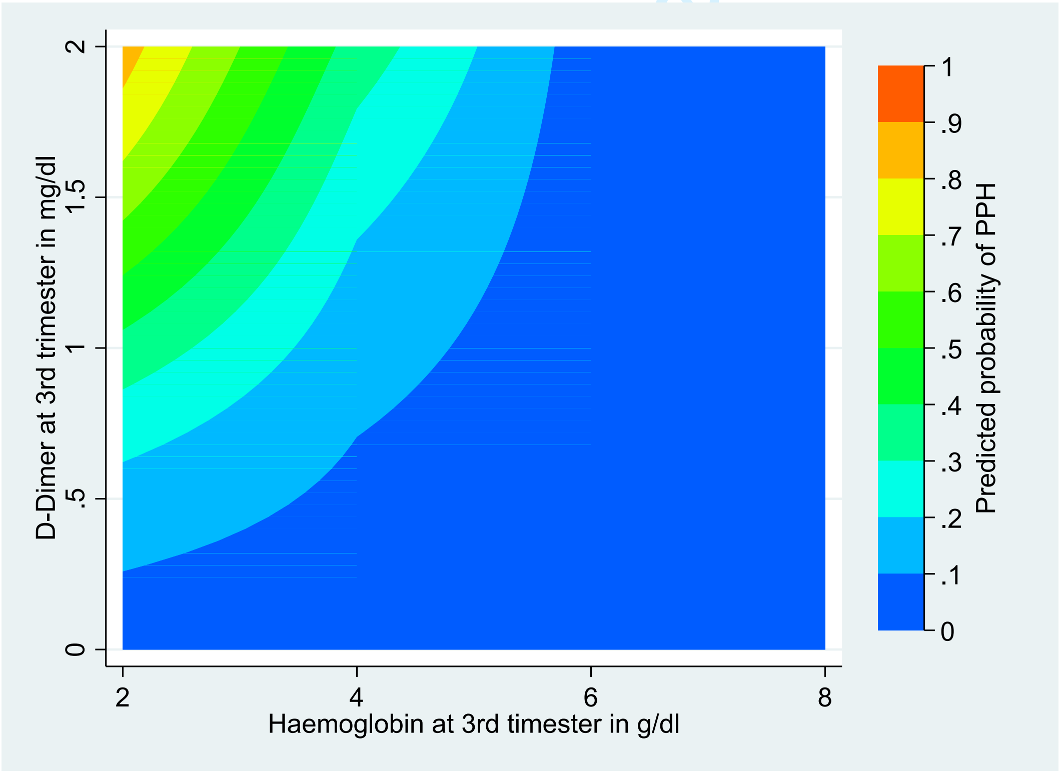

STROBE Statement—Checklist of items that should be included in reports of ***cohort studies***

	Item No	Recommendation	Page No
**Title and abstract**	1	(*a*) Indicate the study's design with a commonly used term in the title or the abstract	1
		(*b*) Provide in the abstract an informative and balanced summary of what was done and what was found	4
**Introduction**			
Background/rationale	2	Explain the scientific background and rationale for the investigation being reported	6
Objectives	3	State specific objectives, including any prespecified hypotheses	6
**Methods**			
Study design	4	Present key elements of study design early in the paper	6
Setting	5	Describe the setting, locations, and relevant dates, including periods of recruitment, exposure, follow-up, and data collection	6,7
Participants	6	(*a*) Give the eligibility criteria, and the sources and methods of selection of participants. Describe methods of follow-up	6,7
		(*b*) For matched studies, give matching criteria and number of exposed and unexposed	N/A
Variables	7	Clearly define all outcomes, exposures, predictors, potential confounders, and effect modifiers. Give diagnostic criteria, if applicable	6,7
Data sources/ measurement	8*	For each variable of interest, give sources of data and details of methods of assessment (measurement). Describe comparability of assessment methods if there is more than one group	6,7
Bias	9	Describe any efforts to address potential sources of bias	7,8
Study size	10	Explain how the study size was arrived at	8
Quantitative variables	11	Explain how quantitative variables were handled in the analyses. If applicable, describe which groupings were chosen and why	6,8
Statistical methods	12	(*a*) Describe all statistical methods, including those used to control for confounding	8
		(*b*) Describe any methods used to examine subgroups and interactions	
		(*c*) Explain how missing data were addressed	
		(*d*) If applicable, explain how loss to follow-up was addressed	
		(*e*) Describe any sensitivity analyses	
**Results**			
Participants	13*	(a) Report numbers of individuals at each stage of study—eg numbers potentially eligible, examined for eligibility, confirmed eligible, included in the study, completing follow-up, and analysed	9,10
		(b) Give reasons for non-participation at each stage	
		(c) Consider use of a flow diagram	
Descriptive data	14*	(a) Give characteristics of study participants (eg demographic, clinical, social) and information on exposures and potential confounders	9,10
		(b) Indicate number of participants with missing data for each variable of interest	
		(c) Summarise follow-up time (eg, average and total amount)	
Outcome data	15*	Report numbers of outcome events or summary measures over time	10

For peer review only

For peer review only - http://bmjopen.bmj.com/site/about/guidelines.xhtml

Main results	16	(*a*) Give unadjusted estimates and, if applicable, confounder-adjusted estimates and their precision (eg, 95% confidence interval). Make clear which confounders were adjusted for and why they were included<br>(*b*) Report category boundaries when continuous variables were categorized<br>(*c*) If relevant, consider translating estimates of relative risk into absolute risk for a meaningful time period	11, 12, 13, 14
Other analyses	17	Report other analyses done—eg analyses of subgroups and interactions, and sensitivity analyses	13
**Discussion**			
Key results	18	Summarise key results with reference to study objectives	14
Limitations	19	Discuss limitations of the study, taking into account sources of potential bias or imprecision. Discuss both direction and magnitude of any potential bias	16
Interpretation	20	Give a cautious overall interpretation of results considering objectives, limitations, multiplicity of analyses, results from similar studies, and other relevant evidence	14, 15
Generalisability	21	Discuss the generalisability (external validity) of the study results	16
**Other information**			
Funding	22	Give the source of funding and the role of the funders for the present study and, if applicable, for the original study on which the present article is based	19

*Give information separately for exposed and unexposed groups.

Note: An Explanation and Elaboration article discusses each checklist item and gives methodological background and published examples of transparent reporting. The STROBE checklist is best used in conjunction with this article (freely available on the Web sites of PLoS Medicine at http://www.plosmedicine.org/, Annals of Internal Medicine at http://www.annals.org/, and Epidemiology at http://www.epidem.com/). Information on the STROBE Initiative is available at http://www.strobe-statement.org.

For peer review only

BMJ Open

Relationship between anaemia, coagulation parameters during pregnancy and postpartum haemorrhage at childbirth: a prospective cohort study

Journal:	*BMJ Open*
Manuscript ID	bmjopen-2021-050815.R1
Article Type:	Original research
Date Submitted by the Author:	26-Jul-2021
Complete List of Authors:	Nair, Manisha; Oxford University, NPEU, Nuffield Department of Population Health<br>Chhabra, Shakuntala ; Mahatma Gandhi Institute of Medical Sciences<br>Choudhury , Saswati; Gauhati Medical College and Hospital<br>Deka, Dipika ; Srimanta Sankaradeva University of Health Sciences<br>Deka, Gitanjali ; Tezpur Medical College<br>Kakoty, Swapna ; Fakhruddin Ali Ahmed Medical College and Hospital<br>Kumar, Pramod ; Mahatma Gandhi Institute of Medical Sciences<br>Mahanta, Pranabika ; Jorhat Medical College and Hospital<br>Medhi, Robin ; Fakhruddin Ali Ahmed Medical College and Hospital<br>Rani, Anjali; Banaras Hindu University Institute of Medical Sciences, Department of Obstetrics and Gynaecology<br>Rao, Seeresha; Silchar Medical College and Hospital<br>Roy, Indrani ; Nazareth Hospital<br>Solomi V, Carolin ; Makunda Christian Leprosy and General Hospital<br>Talukdar, Ratna ; Jorhat Medical College and Hospital<br>Zahir, Farzana; Assam Medical College, Department of Obstetrics and Gynaecology<br>Kansal, Nimmi ; Dr Lal Pathlabs, National Reference Laboratory<br>Arora, Anil; Dr Lal PathLabs, National Reference Laboratory<br>Opondo, Charles; Oxford University, Nuffield Department of Population Health<br>Armitage, Jane; Oxford University, Nuffield Department of Population Health<br>Laffan, Michael; Imperial College London Faculty of Medicine, Haemostasis & Thrombosis<br>Stanworth, Simon; Oxford University, Department of Haematology/Transfusion Medicine<br>Quigley, Maria; Oxford University, National Perinatal Epidemiology Unit<br>Baigent, Colin; Oxford University, Nuffield Department of Population Health<br>Knight, Marian; Oxford University, National Perinatal Epidemiology Unit<br>Kurinczuk, Jennifer; Oxford University, National Perinatal Epidemiology Unit
**Primary Subject Heading**:	Obstetrics and gynaecology
Secondary Subject Heading:	Epidemiology, Global health, Haematology (incl blood transfusion)

Keywords:	Anaemia < HAEMATOLOGY, EPIDEMIOLOGY, OBSTETRICS

SCHOLARONE™
Manuscripts

I, the Submitting Author has the right to grant and does grant on behalf of all authors of the Work (as defined in the below author licence), an exclusive licence and/or a non-exclusive licence for contributions from authors who are: i) UK Crown employees; ii) where BMJ has agreed a CC-BY licence shall apply, and/or iii) in accordance with the terms applicable for US Federal Government officers or employees acting as part of their official duties; on a worldwide, perpetual, irrevocable, royalty-free basis to BMJ Publishing Group Ltd ("BMJ") its licensees and where the relevant Journal is co-owned by BMJ to the co-owners of the Journal, to publish the Work in this journal and any other BMJ products and to exploit all rights, as set out in our licence.

The Submitting Author accepts and understands that any supply made under these terms is made by BMJ to the Submitting Author unless you are acting as an employee on behalf of your employer or a postgraduate student of an affiliated institution which is paying any applicable article publishing charge ("APC") for Open Access articles. Where the Submitting Author wishes to make the Work available on an Open Access basis (and intends to pay the relevant APC), the terms of reuse of such Open Access shall be governed by a Creative Commons licence – details of these licences and which Creative Commons licence will apply to this Work are set out in our licence referred to above.

Other than as permitted in any relevant BMJ Author's Self Archiving Policies, I confirm this Work has not been accepted for publication elsewhere, is not being considered for publication elsewhere and does not duplicate material already published. I confirm all authors consent to publication of this Work and authorise the granting of this licence.

For peer review only

Relationship between anaemia, coagulation parameters during pregnancy and postpartum haemorrhage at childbirth: a prospective cohort study

Authors

1. Manisha Nair, DPhil
National Perinatal Epidemiology Unit, Nuffield Department of Population Health, University of Oxford, Old Road Campus, Headington, Oxford, OX3 7LF, UK
Email: manisha.nair@npeu.ox.ac.uk

2. Shakuntala Chhabra, MD
Mahatma Gandhi Institute of Medical Sciences,
Sevagram, Maharashtra 442102, India
Email: schhabra@mgims.ac.in

3. Saswati S Choudhury, MD
Gauhati Medical College and Hospital (GMCH)
Bhangagarh, Guwahati, Assam 781032, India
Email: saswatischoudhury@gmail.com

4. Dipika Deka, MD
Srimanta Sankaradeva University of Health Sciences,
Narkashur Hilltop, Bhangagarh, Guwahati, Assam 781032, India
Email: dpk.deka@gmail.com

5. Gitanjali Deka, MD
Tezpur Medical College
NH 15, Tezpur, Assam 784153, India
Email: gitanjali.dk@gmail.com

6. Swapna D Kakoty, MD
Fakhruddin Ali Ahmed Medical College and Hospital (FAAMCH)
Barpeta-Hospital-Jania Rd, Joti Gaon, Assam 781301, India
Email: swapna_kakoty@rediffmail.com

7. Pramod Kumar, MD
Mahatma Gandhi Institute of Medical Sciences,
Sevagram, Maharashtra 442102, India
Email: pkumar@mgims.ac.in

8. Pranabika Mahanta, MD
Jorhat Medical College and Hospital
Kushal Konwar Path, Barbheta, Jorhat, Assam 785001, India
Email: drpranabikamahanta@gmail.com

9. Robin Medhi, MD
Fakhruddin Ali Ahmed Medical College and Hospital (FAAMCH)
Barpeta-Hospital-Jania Rd, Joti Gaon, Assam 781301, India
Email: robinmedhi@yahoo.com

For peer review only - http://bmjopen.bmj.com/site/about/guidelines.xhtml

10. Anjali Rani, MD
Institute of Medical Sciences, Banaras Hindu University
Aurobindo Colony, Banaras Hindu University Campus, Varanasi, Uttar Pradesh 221005, India
Email: anjalirani.imsbhu@gmail.com

11. Seeresha Rao, MD
Silchar Medical College and Hospital (SMCH), Ghungoor Road, Masimpur, Uttar Krishnapur Pt III, Assam 788014, India
Email: sereesha2000@gmail.com

12. Indrani Roy, MD
Nazareth Hospital
Arbuthnot Rd, Nongkynrih, Laitumkhrah, Shillong, Meghalaya 793003, India
Email: indraniroydeb@gmail.com

13. Carolin Solomi V, MS
Makunda Christian Leprosy and General Hospital
Bazaricherra, Karimganj, Assam 788727, India
Email: dr_carolin@rediffmail.com

14. Ratna Kanta Talukdar, MD
Jorhat Medical College and Hospital
Kushal Konwar Path, Barbheta, Jorhat, Assam 785001, India
Email: rktalukdar@gmail.com

15. Farzana Zahir, MD
Assam Medical College (AMC)
Barbari, Dibrugarh, Assam 786002, India
Email: sajeedfarah@hotmail.com

16. Nimmi Kansal, MD
National Reference Laboratory, Dr Lal PathLabs
B7 Rd, Block E, Sector 18, Rohini, New Delhi, Delhi 110085, India
Email: nimmi.kansal@lalpathlabs.com

17. Anil Arora, MD
National Reference Laboratory, Dr Lal PathLabs
B7 Rd, Block E, Sector 18, Rohini, New Delhi, Delhi 110085, India
Email: Anil.Arora@lalpathlabs.com

18. Charles Opondo, PhD
National Perinatal Epidemiology Unit, Nuffield Department of Population Health, University of Oxford, Old Road Campus, Headington, Oxford, OX3 7LF, UK
Email: charles.opondo@npeu.ox.ac.uk

19. Jane Armitage, MD
Clinical Trial Service Unit, Nuffield Department of Population Health, University of Oxford, Old Road Campus, Headington, Oxford, OX3 7LF, UK

For peer review only - http://bmjopen.bmj.com/site/about/guidelines.xhtml

Email: jane.armitage@ctsu.ox.ac.uk

20. Michael A Laffan, FRCPath
Department of Haematology, Faculty of Medicine at Imperial College, Hammersmith Hospital, London, W12 0HS, UK
Email: m.laffan@imperial.ac.uk

21. Simon J Stanworth, FRCPath
Department of Haematology, NHS Blood and Transplant/Oxford University Hospitals NHS Foundation Trust, John Radcliffe Hospital; Radcliffe Department of Medicine, University of Oxford, Oxford, OX3 9DU, UK
Email: simon.stanworth@nhsbt.nhs.uk

22. Maria Quigley, MSc
National Perinatal Epidemiology Unit, Nuffield Department of Population Health, University of Oxford, Old Road Campus, Headington, Oxford, OX3 7LF, UK
Email: maria.quigley@npeu.ox.ac.uk

23. Colin Baigent, FMedSci
MRC Population Health Research Unit, Nuffield Department of Population Health, University of Oxford, Old Road Campus, Headington, Oxford, OX3 7LF, UK
Email: colin.baigent@ndph.ox.ac.uk

24. Marian Knight, FRCPE
National Perinatal Epidemiology Unit, Nuffield Department of Population Health, University of Oxford, Old Road Campus, Headington, Oxford, OX3 7LF, UK
Email: marian.knight@npeu.ox.ac.uk

25. Jenny J Kurinczuk, FFPH
National Perinatal Epidemiology Unit, Nuffield Department of Population Health, University of Oxford, Old Road Campus, Headington, Oxford, OX3 7LF, UK
Email: jenny.kurinczuk@npeu.ox.ac.uk

on behalf of the MaatHRI collaborators^

Authors 2-15 contributed equally

^Details about the MaatHRI collaboration can be found on https://www.npeu.ox.ac.uk/maathri

Corresponding author

Manisha Nair
National Perinatal Epidemiology Unit, Nuffield Department of Population Health, University of Oxford, Old Road Campus, Headington, Oxford, OX3 7LF, UK
Email: manisha.nair@npeu.ox.ac.uk; Phone: 01865 617820

For peer review only

Abstract

Objectives: To investigate the association between coagulation parameters and severity of anaemia (moderate anaemia: haemoglobin (Hb) 7-9.9g/dl and severe anaemia: Hb<7g/dl) during pregnancy and relate these to postpartum haemorrhage (PPH) at childbirth.

Design: A prospective cohort study of pregnant women recruited in the third trimester and followed-up after childbirth.

Setting: Ten hospitals across four states in India.

Participants: 1342 pregnant women

Intervention: Not applicable

Methods: Hb and coagulation parameters: fibrinogen, D-dimer, D-dimer/fibrinogen ratio, platelets, and INR (International normalised ratio) were measured at baseline. Participants were followed-up to measure blood loss within two-hours after childbirth and PPH was defined based on blood loss and clinical assessment. Associations between coagulation parameters, Hb, anaemia and PPH were examined using multivariable logistic regression models.

Outcomes measures: Adjusted Odds Ratio (aOR) with 95% confidence interval(CI).

Results: In women with severe anaemia during the third trimester, the D-dimer was 27% higher, mean fibrinogen 117mg/dl lower, D-dimer/fibrinogen ratio 69% higher, and INR 12% higher compared to women with no/mild anaemia. Mean platelets in severe anaemia was $37.8X10^9$/L lower compared with women with moderate anaemia. Similar relationships with smaller effect sizes were identified for women with moderate anaemia compared with women with no/mild anaemia. Low Hb and high INR at third trimester of pregnancy independently increased the odds of PPH at childbirth, but the other coagulation parameters were not found to be significantly associated with PPH.

Conclusion: Altered blood coagulation profile in pregnant women with severe anaemia could be a risk factor for PPH and requires further evaluation.

ARTICLE SUMMARY

Strengths and limitations of this study

- This is the first study to investigate the role of coagulation in relation to the increased risk of PPH in women with moderate/ severe anaemia.
- The large prospective cohort study substantially removed the potential for reverse causation when estimating the effect of the coagulation parameters on PPH.
- Another strength is reproducibility because we examined the relationship of Hb with five different parameters of coagulation and all suggested a similar effect.

For peer review only

- The follow-up rate was 88% and the mean Hb concentration at baseline (exposure of interest) for 12% participants who could not be followed-up was not different from the participants who were followed-up.

Key words: anaemia, coagulation parameters, pregnancy, postpartum haemorrhage, cohort study

Word count: 3955

For peer review only

For peer review only - http://bmjopen.bmj.com/site/about/guidelines.xhtml

INTRODUCTION

Moderate (Hb7-9.9g/dl[1]) and severe anaemia (Hb<7g/dl[1]) during pregnancy not only increase the risk of postpartum haemorrhage (PPH), but also increase the risk of dying from PPH by several fold[2-4]. The public health problem of anaemia during pregnancy is graded as moderate-severe in 183 countries across the world with about 32.4 million (95% CI: 28.4—36.2) pregnant women with anaemia in 2011, globally[5]. Explanations include low Hb associated with reduced oxygen availability resulting in reduced uterine contractility and early fatigue causing uterine atony and PPH[6]. However, changes in the coagulation profile in anaemic pregnant women may also predispose them to an increased risk of bleeding.

Pregnancy is a state of physiological hypercoagulability, with an increase in fibrinogen and decrease in fibrinolytic activity, with increasing gestational age[7 8]. An expansion in plasma volume results in a physiological decrease in platelets, haematocrit and Hb during pregnancy[7], although the prothrombin time remains largely stable[7 9 10]. While the haemostatic changes in normal pregnancy are well described, there have been few investigations of the relationship and potential clinical implications of coagulation abnormalities in association with severe anaemia in pregnant women. D-dimer was shown to be useful in risk stratification to rule out pulmonary embolism and to limit exposure of suspected pregnant women to imaging[11], but the role of D-dimer and other coagulation parameters in risk stratification of PPH is not clear.

The primary objective of this study was to investigate the association between blood coagulation parameters (fibrinogen, D-dimer, D-dimer/fibrinogen ratio, platelets and INR (International normalised ratio)) and severity of anaemia during the third trimester of pregnancy. The secondary objective was to examine the relationship between anaemia and coagulation parameters during the third trimester and PPH at childbirth.

METHODS

Study design

A hospital-based prospective cohort study undertaken through the Maternal and perinatal Health Research collaboration, India (MaatHRI)[12].

Study population

All pregnant women >28 weeks of gestation, ≥18 years, and planning a vaginal birth in 10 MaatHRI collaborating hospitals across four states in India (Assam, Meghalaya, Uttar Pradesh and Maharashtra) were approached to participate in the study. The response rate was 99.8% and 1342 eligible pregnant women who provided written informed consent were recruited between October 2018 and May 2019. The women were followed-up during labour and childbirth and up to 48 hours postpartum.

Baseline data

Information was collected from women during the baseline assessment about sociodemographic characteristics, previous and current pregnancy problems, medical co-morbidities, and other pregnancy characteristics. Blood samples were collected to measure Hb, haematocrit, fibrinogen, D-dimer, platelets, prothrombin time (from which INR was derived), and if present, cause of anaemia (inferred from measurement of serum ferritin and Hb electrophoresis). Using the WHO definition for anaemia in pregnancy[1], women with Hb ≥10g/dl were classified as no/mild anaemia, 7-9.9g/dl as moderate and <7g/dl as severe anaemia. We generated a D-dimer/fibrinogen ratio that was used in other studies[8 13 14]. Since

For peer review only - http://bmjopen.bmj.com/site/about/guidelines.xhtml

INR is not influenced by the pregnancy state[9 10], using the standard cut-off, we classified pregnant women into high (>1.1) and low INR (≤1.1) groups. Other coagulation parameters were analysed as continuous variables.

Laboratory methods

The MaatHRI platform has a laboratory infrastructure through a partnership with a private laboratory in India[12]. Blood collection, processing, storage and analysis were standardised. All blood samples were analysed at the national laboratory. The assay methods, traceability and performance characteristics for each test were agreed with experts from the University of Oxford's Wolfson laboratory and the Indian laboratory partner. Supplementary Table S1 shows the traceability and in Table S2 we present the assay methods and their performance characteristics.

The laboratory measured time in transit for each sample and their quality. Depending upon the remoteness of the hospital, the transit time ranged between 12 and 72 hours. Three types of samples were collected: EDTA whole blood for Hb, haematocrit, platelets and Hb electrophoresis, serum for ferritin, and citrated plasma for D-dimer, fibrinogen and INR. Citrated plasma samples were centrifuged at 3700 revolutions per minute (RPM) for 10 minutes using centrifuge machines of same make and model in all study hospitals, and aliquot was prepared from supernatant plasma, frozen immediately and shipped with dry ice. Samples that were inadequate, in terms of quantity, stability, temperature and other quality indicators, were discarded. A variable, 'hospital-code', was generated to account for transit time and other known and unknown potential biases related to sample quality in the statistical analysis.

Follow-up data

Similar to other studies[15 16], a calibrated blood collection drape was used to objectively measure the amount of blood loss within two hours after childbirth. The same make and model of drape (PPH alert bag) was used in all study hospitals. The drape was placed immediately after the birth of the baby (before removing the placenta) and blood loss was measured from the calibrated and colour coded markings on the drape. While maximum blood loss is just before and after the removal of placenta and up to one hour after childbirth, the drape was left in situ up to two hours during the post-birth observation period in the labour room if the woman continued to bleed[16]. The drape could not be used for women who had a caesarean section, in which case estimates of blood loss were measured by the obstetrician from the suction bottle and soaked sponges. Only pregnant women with a planned vaginal birth were recruited in the study, thus the participants who had a caesarean section were women who had an emergency section after spontaneous rupture of membranes. As a result, the suction bottle contained very little liquor thereby making the blood loss estimates more accurate. The objective measurement methods were in line with the recommendations of the American College of Obstetricians and Gynecologists (ACOG)[17]. ACOG acknowledges the difficulty in accurately measuring blood loss after childbirth, but recommends use of calibrated drapes and hospital-based protocols for collecting and measuring blood loss after childbirth, which are more accurate than visual estimation[17].

PPH was defined based on measured blood loss within two hours after childbirth (≥500ml for women who had a vaginal birth and ≥1000ml for women who had a caesarean birth) and clinician diagnosed PPH requiring management. This was similar to the methods used to define PPH in other studies[15 16]. We also collected information about the mode of birth, maternal complications at birth, admission to intensive care unit and maternal death.

For peer review only - http://bmjopen.bmj.com/site/about/guidelines.xhtml

Sample size

A priori sample size calculations were done for two primary parameters: D-dimer and fibrinogen (see Table-S3). Sample sizes were calculated for a range of expected changes in the mean concentrations of the parameters (10%, 20% and 30%) between the no/mild anaemia and moderate/severe anaemia groups taking power (1-β)=90%, α=5% (two-tailed), and n1=n2. A sample size of 1028 had adequate power to detect a mean difference of 10% in the concentration of D-dimer and fibrinogen between the two study groups assuming a mean of 0.11 mg/dl (SD=0.573) for D-dimer[18] and 379 mg/dl (SD=0.78) for fibrinogen[19] in the baseline groups. This was inflated by 15% to account for potential loses which led to a total sample of 1209, rounded off to 1200 (n1=n2=600). However, we were able to increase the sample size during the recruitment phase, finally recruiting 1342 pregnant women, which allowed us to examine the difference in the concentration of coagulation parameters between three groups: no/mild anaemia, moderate and severe anaemia.

Statistical analysis

Descriptive statistics were used for all blood parameters, participant characteristics at baseline and PPH at childbirth. We calculated and compared the mean Hb across categories of gestational age, and mean gestational age across the categories of anaemia using t-test with Bartlett's statistics for equal variances. We examined the distribution of the continuous variables, and blood parameters that were not normally distributed: D-dimer, D-dimer/fibrinogen ratio and INR, were log transformed to create a normal distribution. These were used as outcome variables in the primary analysis and multivariable linear regression models were used to examine their individual association with Hb and anaemia after controlling for known confounding variables including gestational age, maternal age, hypertensive disorders of pregnancy (which included gestational hypertension, pre-eclampsia, eclampsia, superimposed pre-eclampsia on chronic hypertension as well as severe forms of pre-eclampsia such as HELLP syndrome), pre-existing medical problems and hospital-code. We conducted tests for linear trend and used Chi-square tests to assess heterogeneity in odds ratios across categories of anaemia. We also examined the presence of any non-linear relationships between Hb and the coagulation parameters. The analysis was repeated using haematocrit instead of Hb as the exposure variable to test reproducibility of the results.

For the secondary objective, we analysed the association of PPH with Hb, anaemia and the coagulation parameters using multivariable logistic regression analysis controlling for potential confounders and exploring significant interactions. We found the variable ‘hospital-code’ to be strongly correlated with hypertensive disorders of pregnancy and PPH. To improve model parsimony, ‘hospital-code’ was not included in the multivariable analysis. To understand whether the effect of Hb on PPH was moderated or mediated by each of the coagulation parameters, we tested for interaction and conducted mediation analysis, respectively. Likelihood-ratio test was used to examine statistically significant interactions at $p<0.1$ considering a lower power for the sub-group analysis. Mediation analysis was undertaken using the generalisation to the Baron-Kenny approach to mediation analysis.[20]

Missing data for the blood parameters were related to samples being discarded due to quality issues, but not with the level of the parameter itself or Hb. Therefore, data in the study were considered missing at random (MAR) and complete case analysis was used. All results were

For peer review only - http://bmjopen.bmj.com/site/about/guidelines.xhtml

considered significant at a two-tailed p-value of <0.05. Analyses were undertaken using Stata version 16, Special Edition (StataCorp, College Station, Texas, USA).

Patient and public involvement

Patient and public were not involved in the design, conduct or reporting of the study.

Ethics approval and participant consent

The study was approved by the institutional review boards (IRB) of each coordinating Indian institution, namely: Srimanta Sankaradeva University of Health Sciences, Guwahati, Assam; Nazareth hospital, Shillong, Meghalaya; Emmanuel Hospital Association, New Delhi; Mahatma Gandhi Institute of Medical Sciences, Sevagram, Maharashtra; and the Institute of Medical Sciences, Banaras Hindu University, Varanasi, Uttar Pradesh. It also received approval from the Government of India's Health Ministry's Screening Committee, the Indian Council of Medical Research, New Delhi and by the Oxford Tropical Research Ethics Committee (OxTREC), University of Oxford, UK.

Written informed consent was taken from all participants.

RESULTS

The baseline characteristics of the study population are presented in Table-1. The mean Hb was 10.3g/dl and mean gestational age at recruitment was 35.3 weeks. Mean Hb was similar in the different periods of gestational age (p=0.275, see Table-1) and gestational age was also similar across the categories of anaemia (p=0.128, see Table-1). The proportions of no/mild, moderate and severe anaemia in the study population were 58.9%, 34.7% and 5.3%, respectively. The most common anaemia was iron deficiency (microcytic-hypochromic anaemia 19.8% and about 28.5% had serum ferritin <15 µg/L), but 12.5% of women had macrocytic anaemia and 14% had an HbE trait or disease. The mean haematocrit was 21% in women with severe anaemia compared with 30% in women with moderate anaemia and 37% in women with mild/no anaemia. About 17% of the study population reported a problem during the current pregnancy. A total of eight women reported an antepartum haemorrhage, of these four were in the category of no/mild anaemia, three in moderate and one in the severe anaemia group.

Table-1: Baseline characteristics of the study population

For peer review only

**Characteristics at baseline (Total participants at baseline)**	Overall study population	No/mild anaemia (Hb ≥10g/dl) N=790	Moderate anaemia (Hb 7-9.9g/dl) N=465	Severe anaemia (Hb<7g/dl) N=71	Missing Hb information N=16
	**Mean (SD)**				
Maternal age (in years); N=1334	24.5 (4.2)	24.5 (4.0)	24.6 (4.4)	24.9 (4.5)	24.2 (4.5)
Gestational age at baseline recruitment (in weeks); N=1342	35.3 (3.7)	35.4 (3.7)	35.1 (3.8)	35.0 (3.7)	34.9 (3.2)
**Blood parameters at baseline (unit of measure)**	**Mean (SD)**				
Hb (Hb in g/dl); N=1326	10.3 (1.9)	-	-	-	-
Hb in g/dl by categories of gestational age (p=0.275)					-
28-32 weeks	10.0 (1.8)	-	-	-	-
33-36 weeks	10.5 (1.9)	-	-	-	-
≥37weeks	10.4 (1.9)	-	-	-	-
Platelets (X 10^9/L); N=1305	195.6 (73.7)	187.4 (2.5)	213.5 (3.6)	168.3 (9.4)	-
Fibrinogen (mg/dl); N=1270	410.9 (129.2)	431.1 (4.8)	390.5 (5.7)	319.9 (12.7)	339.1 (98.6)
	**Median (IQR)**				
D-dimer (mg/dl); N=1264	0.08 (0.07)	0.07 (0.06)	0.08 (0.07)	0.10 (0.09)	0.12 (0.13)
D-dimer/fibrinogen ratio; N=1250	0.0002 (.0002)	0.0002 (0.0002)	0.0002 (0.0002)	0.0003 (0.0005)	0.0005 (0.0005)
International normalised ratio (INR); N=1243	0.96 (0.12)	0.94 (0.13)	0.96 (0.1)	0.99 (0.13)	0.96 (0.31)
Body mass index (BMI) at first antenatal check-up (kg/m^2); N= 1165	21.1 (4.3)	21.2 (4.6)	20.8 (4.4)	21.1 (4.2)	21.2 (3.8)
	**No. of women (%)**				
INR					
≤1.1	1126 (83.9)	679 (86.0)	394 (84.7)	49 (69.0)	4 (25.0)
>1.1	117 (8.7)	61 (7.7)	40 (8.6)	14 (19.7)	2 (12.5)
Missing	99 (7.4)	50 (6.3)	31 (6.7)	8 (11.3)	10 (62.5)
HbE					
Normal	1141 (85.0)	689 (87.2)	390 (83.9)	62 (87.3)	0 (0)
Trait	129 (9.6)	84 (10.6)	38 (8.2)	7 (9.9)	0 (0)
Disease	56 (4.2)	17 (2.2)	37 (7.9)	2 (2.8)	0 (0)

For peer review only - http://bmjopen.bmj.com/site/about/guidelines.xhtml

Missing	16 (1.2)	0 (0)	0 (0)	0 (0)	16 (100.0)
Microcytic-hypochromic anaemia					
No	1010 (75.3)	684 (86.6)	293 (63.0)	33 (46.5)	0 (0)
Yes	266 (19.8)	84 (10.6)	155 (33.3)	27 (38.0)	0 (0)
Missing	66 (4.9)	22 (2.8)	17 (3.7)	11 (15.5)	16 (100.0)
Macrocytic anaemia					
No	1110 (82.7)	647 (81.9)	413 (88.8)	50 (70.4)	0 (0)
Yes	168 (12.5)	121 (15.3)	36 (7.7)	11 (15.5)	0 (0)
Missing	64 (4.8)	22 (2.8)	16 (3.4)	10 (14.1)	16 (100.0)
Serum Ferritin					
≥ 15 µg/L	927 (69.1)	612 (77.5)	269 (57.8)	39 (54.9)	7 (43.7)
< 15 µg/L	383 (28.5)	165 (20.9)	190 (40.9)	28 (39.4)	0 (0)
Missing	32 (2.4)	13 (1.6)	6 (1.3)	4 (5.6)	9 (56.3)
**Other pregnancy characteristics at baseline**					
Hypertensive disorders of pregnancy					
No	1290 (96.1)	761 (96.3)	447 (96.1)	66 (93.0)	16 (100)
Yes	46 (3.4)	24 (3.0)	17 (3.7)	5 (7.0)	0 (0)
Missing	6 (0.5)	5 (0.6)	1 (0.2)	0 (0)	0 (0)
Pre-existing medical problems (other than haemoglobinopathies)					
No	1281 (95.5)	759 (96.1)	443 (95.3)	63 (88.7)	16 (100)
Yes	58 (4.3)	29 (3.7)	21 (4.5)	8 (11.3)	0 (0)
Missing	3 (0.2)	2 (0.2)	1 (0.2)	0 (0)	0 (0)

Pre-existing medical problems (excluding haemoglobinopathies) included diabetes, essential hypertension, rheumatic heart disease, hypothyroidism, urinary tract infection, kidney stone, appendicitis, gall bladder problems, ovarian tumour, pulmonary tuberculosis and Hepatitis C infection.

For peer review only - http://bmjopen.bmj.com/site/about/guidelines.xhtml

Key follow-up data are presented in Table-2. There was a 12% loss to follow-up, but no difference in mean Hb during the third trimester between women who were followed-up (10g/dl) and those not followed-up (10g/dl). A flow chart showing the study population is provided in Figure-S1.

Table-2: Key data from the follow-up

**Follow-up (Total participants at follow-up=1178, 12% loss to follow-up)**	Overall study population followed-up N=1178	No/mild anaemia (Hb ≥10g/dl) N=709	Moderate anaemia (Hb 7-9.9g/dl) N=390	Severe anaemia (Hb<7g/dl) N=66	Missing Hb information N=13
**PPH**	**No. of women (%)**				
No	1159 (98.4)	701 (98.9)	382 (97.9)	63 (95.5)	13 (100)
Yes	19 (1.6)	8 (1.1)	8 (2.1)	3 (4.5)	0 (0)
**Mode of delivery**					
Vaginal birth	852 (72.3)	515 (72.6)	279 (71.4)	49 (74.2)	9 (69.2)
Caesarean birth	326 (27.7)	194 (27.4)	111 (28.5)	17 (25.8)	4 (30.8)

PPH-postpartum haemorrhage

Association of coagulation parameters with Hb and anaemia

The results of the linear regression analyses are presented in Table-3 and Figures 1-6. All coagulation parameters were significantly associated with Hb and anaemia during the third trimester. The relationships were linear (inverse linear associations), except for platelets that had a non-linear inverted J-shaped association with Hb (Figure-1).

Table-3: Association of coagulation parameters with Hb and anaemia at third trimester

	**Outcome variables**		
	**D-dimer**		
**Independent variables**	Unadjusted Coefficient (95% CI)	Adjusted* Coefficient (95% CI)	P value - test for linear trend
Hb	0.96 (0.94 to 0.97)	0.96 (0.94 to 0.98)	<0.001
No/mild anaemia	1 (ref)[§]	1 (ref)[§]	0.003[£]
Moderate anaemia	1.11 (1.02 to 1.20)	1.08 (0.99 to 1.17)	
Severe anaemia	1.25 (1.04 to 1.50)	1.27 (1.07 to 1.50)	
	**Fibrinogen**		
	Unadjusted Coefficient (95% CI)	Adjusted* Coefficient (95% CI)	P value - test for linear trend
Hb	14.68 (11.11 to 18.24)	15.58 (12.08 to 19.09)	<0.001
No/mild anaemia	0 (Ref)	0 (Ref)	<0.001[£]
Moderate anaemia	-40.5 (-55.3 to -25.7)	-39.2 (-53.7 to -24.9)	
Severe anaemia	-111.1 (-143.4 to -78.9)	-117.2 (-148.3 to -86.1)	

For peer review only - http://bmjopen.bmj.com/site/about/guidelines.xhtml

	D-dimer/fibrinogen ratio		
	Unadjusted Coefficient (95% CI)	Adjusted* Coefficient (95% CI)	P value - test for linear trend
Hb	0.92 (0.90 to 0.95)	0.93 (0.91 to 0.95)	<0.001
No/mild anaemia	1 (ref)[§]	1 (ref)[§]	<0.001[£]
Moderate anaemia	1.21 (1.09 to 1.34)	1.17 (1.06 to 1.29)	
Severe anaemia	1.63 (1.30 to 2.06)	1.69 (1.36 to 2.09)	
	**INR**		
	Unadjusted Coefficient (95% CI)	Adjusted* Coefficient (95% CI)	P value - test for linear trend
Hb	0.98 (0.97 to 0.99)	0.99 (0.98 to 0.99)	0.001
No/mild anaemia	1 (ref)[§]	1 (ref)[§]	0.007[£]
Moderate anaemia	1.03 (0.99 to 1.06)	1.02 (0.98 to 1.05)	
Severe anaemia	1.09 (1.02 to 1.17)	1.12 (1.04 to 1.19)	
	**Platelets**		
	Unadjusted Coefficient (95% CI)	Adjusted* Coefficient (95% CI)	P value - test for linear trend
Hb	-3.79 (-5.83 to -1.74)	-4.57 (-6.64 to -2.49)	NA
No/mild anaemia	-26.04 (-34.40 to -17.68)	-26.09 (-34.51 to -17.67)	NA
Moderate anaemia	0 (ref)	0 (ref)	
Severe anaemia	-45.21 (-63.91 to -26.51)	-37.78 (-56.47 to -19.09)	

*Adjusted for gestational age, maternal age, hypertensive disorders of pregnancy, pre-existing medical problems and hospital-code; [§]Exponent of Log, hence reference is '1' (instead of zero); NA – not applicable; [£]P value - test for linear trend

After adjustment, the D-dimer concentration was 8% (95%CI -1 to +17%) higher in women with moderate anaemia and 27% (95% CI 7 to 50%) higher in severe anaemia compared with no/mild anaemia (p-value for linear trend=0.003). In women with moderate anaemia, the mean fibrinogen concentration was 39.2mg/dl (95% CI 24–53.7.9mg/dl) lower, and in severe anaemia 117.2mg/dl (95% CI 86.1–148.3mg/dl) lower than in women with no/mild anaemia (p-value for trend <0.001). Consequently, the D-dimer/fibrinogen ratio was 17% (95% CI 6–29%) and 69% (95% CI 36–100%) higher respectively in women with moderate and severe anaemia compared with women with no/mild anaemia (p-value for trend<0.001).

Given the inverted J-shaped association between Hb and platelets, the moderate anaemia group was taken as the comparator. Compared to women with moderate anaemia, those with no/mild anaemia had a mean platelet concentration 26 x 10^9/L (95% CI 17.7 to 34.5 x 10^9/L) lower, and those with severe anaemia 38 x 10^9/L (19.1 to 56.5 x 10^9/L) lower. The INR was 2% (95% CI -2 to 5%) and 12% (95% CI 4 to 19%) higher in women with moderate and severe anaemia respectively compared with women with no/mild anaemia. The odds of having a high INR (>1.1) decreased by 19% per g/dl increase in Hb (adjusted OR (aOR) 0.81, 95% CI 0.73 to 0.91, p<0.001). The odds of having a high INR in women with moderate anaemia was not significantly different from women with no/mild anaemia (aOR 1.12, 95% CI 0.69 to 1.84, p=0.647), but women with severe anaemia had more than five-fold higher odds of having a high INR (aOR 5.10, 95% CI 2.31 to 11.29, p<0.001).

For peer review only - http://bmjopen.bmj.com/site/about/guidelines.xhtml

The tests for heterogeneity showed that all odds ratios were significantly different across the categories of anaemia. Figures 2 to 6 show the relationship between the coagulation parameters and the categories of anaemia. The findings did not change when stratified by types of anaemia, although the 95% CI widened due to the small numbers in each stratified category. Furthermore, repeating the analyses using haematocrit as the exposure variable did not change the results materially (Table-S4).

Association of PPH at childbirth with Hb and anaemia in the third trimester of pregnancy

After adjusting for known confounders, the odds of having a PPH at childbirth *increased* by 22% per g/dl *decrease* in Hb (aOR 0.78, 95% CI 0.63 to 0.98). The adjusted odds of having a PPH was nearly two-fold higher in women with moderate anaemia and more than five-fold higher in women with severe anaemia compared with women with mild/no anaemia. There was a significant linear trend of increasing adjusted odds of PPH with increasing severity of anaemia (p-value for linear trend 0.035) (Table-4).

Table-4: Association of PPH at childbirth with Hb and anaemia

**Independent variables**	**Outcome: PPH at childbirth**		
	Unadjusted OR (95% CI)	Adjusted* OR (95% CI)	P value – test for linear trend
Anaemia			
No/mild	1 (ref)	1 (ref)	0.035
Moderate	1.84 (0.68 to 4.93)	1.82 (0.66 to 5.01)	
Severe	4.17 (1.08 to 16.12)	5.11 (1.19 to 21.93)	

*Adjusted for gestational age, maternal age, hypertensive disorders of pregnancy, pre-existing medical problems and mode of birth

Association of PPH at childbirth with coagulation parameters in the third trimester of pregnancy

After adjusting for confounders, the odds of having a PPH increased by more than five-fold in women who had an INR >1.1 during the third trimester of pregnancy (Table-5). The other coagulation parameters, D-dimer, fibrinogen and platelets were not significantly associated with PPH at childbirth (Table-5). Mediation analysis showed no significant mediation of the effect of Hb on PPH via any coagulation parameter. There was a pattern of increasing predicted probability of PPH with a decrease in Hb and increase in D-dimer (Figure S2). Nevertheless we did not find evidence of statistical interaction between Hb and D-dimer in their association with PPH (p-value 0.529). We did not find any significant interaction between Hb and the other coagulation parameters.

Table-5: Association between PPH at childbirth and coagulation parameters in the third trimester of pregnancy

For peer review only - http://bmjopen.bmj.com/site/about/guidelines.xhtml

Outcome- PPH at childbirth	Predictors - Coagulation parameters in the third trimester of pregnancy	
	Unadjusted OR (95% CI)	Adjusted* OR (95% CI)
	D-dimer	
No	1 (ref)	1 (ref)
Yes	1.00 (0.79 to 1.26)	1.03 (0.80 to 1.32)
	Fibrinogen	
No	1 (ref)	1 (ref)
Yes	1.00 (0.99 to 1.006)	1.00 (0.99 to 1.005)
	Platelets	
No	1 (ref)	1 (ref)
Yes	0.99 (0.98 to 1.001)	0.99 (0.98 to 1.002)
	INR >1.1	
No	1 (ref)	1 (ref)
Yes	1.76 (0.39 to 7.78)	5.74 (1.09 to 30.19)

*Regression models adjusted for gestational age, maternal age, hypertensive disorders of pregnancy, pre-existing medical problems and mode of delivery; D-dimer in mg/L FEU; Fibrinogen in mg/dl; platelets x10^9/L

DISCUSSION

The study showed that pregnant women with severe anaemia during the third trimester of pregnancy had a higher D-dimer, lower fibrinogen and therefore a higher D-dimer/fibrinogen ratio than those with mild or moderate anaemia as well as a higher INR, after controlling for known confounders. Similar associations were observed among women with moderate anaemia with levels intermediate between severe and mild anaemia. Having a lower Hb and high INR (>1.1) during the third trimester of pregnancy was independently associated with higher odds of PPH, but we did not find any association between PPH and the other coagulation parameters.

Studies[2 21], including our previous study in India[3], have repeatedly shown that pregnant women with anaemia (particularly severe anaemia) are at a higher risk of PPH. It is also known that low fibrinogen, high INR, and high D-dimer or other measures of fibrinolysis are associated with an increased risk of PPH[7] but to what extent these changes are associated with anaemia has not previously been described. The association between platelets and PPH is not clear[7], nor whether it is the total concentration or functionality of platelets that matter[7].

This study identified a new potential role of an impaired coagulation profile in pregnant women with anaemia that could lead to PPH. These potential associations are hypothesis generating for further research, both to understand the direct causal effects and the mechanisms by which the coagulation changes might exert an impact on anaemic women at childbirth. The primary observation was lower fibrinogen level in women with moderate and severe anaemia in the study population. It is known that fibrinogen levels increase by more than 200% during pregnancy[7] compared to the non-pregnant state to prevent haemorrhage during childbirth, and a recent meta-analysis of concentration of coagulation parameters by gestational age in pregnancy estimated a mean fibrinogen level of 556 (466-664) mg/dl during the third trimester of pregnancy[22]. Compared with this, mean fibrinogen levels during the third trimester were

For peer review only - http://bmjopen.bmj.com/site/about/guidelines.xhtml

391(379-402) mg/dl and 320(295-345) mg/dl in pregnant women with moderate and severe anaemia, respectively in the study population with a linear decrease in fibrinogen level by severity of anaemia, thus potentially increasing the risk of PPH.

There is some evidence that haemodilution has a profibrinolytic effect[23 24], thus another possibility is the presence of low grade pre-delivery fibrinolysis in pregnant women with severe anaemia in the study, which might also predispose them to higher blood loss or haemorrhage at childbirth. The median D-dimer levels in the study population in different categories of anaemia (Table-3) was comparable with the estimated mean D-Dimer during the third trimester of pregnancy in the meta-analysis[22], but we found a linear increase in D-dimer-to-fibrinogen ratio with increase in severity of anaemia. Under a conventional state of hypercoagulability during pregnancy, the decrease in fibrinogen should have been matched with a decrease in fibrinolytic activity, but in our study population with moderate and severe anaemia, the two processes seem to be operating in opposite directions, thereby creating a potential imbalance in clot formation and lysis which could increase the risk of PPH. Further, we also observed a pattern of low Hb and high D-dimer having a multiplicative effect on increased probability of PPH, although the interaction was not statistically significant. We did not find any underlying cause of blood loss (example placenta praevia or abruption), or antepartum haemorrhage in pregnant women with anaemia that could explain both low Hb and high D-dimer.

Likewise, the relative increase in INR in pregnant women with severe anaemia cannot be explained by the physiological changes in pregnancy as INR generally remains stable in pregnancy[10]. Women with severe anaemia had a low haematocrit (21%). While high haematocrit (>50%) is thought to artificially prolong PT from which INR is calculated, a low haematocrit (<25%) should not affect the measurement of PT using standard sodium citrate tubes[25]. It is possible that women with severe anaemia, who were mostly iron deficient, also have vitamin K deficiency due to malnutrition leading to an increase in INR. Prolongation of PT and increase in INR have been shown in patients with sickle cell disease, the increase being proportional to the severity of anaemia[26], and in a study of patients with haematological malignancies who were treated with chemotherapy[27], suggesting a delay in the initiation of the coagulation cascade in people with low Hb. This could explain the observed higher odds of PPH associated with high INR >1.1 in our study population.

We found an inverted J-shaped association between platelets and severity of anaemia. While the lower mean concentration of platelets in women with severe anaemia is in line with the impairment in the other coagulation parameters, the reasons for the lower mean concentration in no/mild anaemia compared with moderate anaemia is unclear. One possible explanation could be residual confounding by hypertensive disorders of pregnancy. Women who have a severe disorder [e.g. HELLP (haemolysis, elevated liver enzymes, and low platelet count) syndrome] have low haemodilution (high Hb) and low platelets[28]. The relationship between anaemia and platelets is also unclear. *In vitro* studies show agglutination of platelets with lowering of Hb[29 30], others found an association between iron deficiency anaemia and thrombocytosis[31 32], and yet others suggest that anaemia impairs the role of red blood cells that normally push the platelets towards the vessel wall during the coagulation process to initiate clot formation[27 33].

Strengths and limitations

For peer review only - http://bmjopen.bmj.com/site/about/guidelines.xhtml

The main strength of this study is that it was large and prospective allowing examination of the relationship between Hb, anaemia and coagulation parameters during late pregnancy and their subsequent effects on blood loss at childbirth. Robust and standardised methods were employed to minimise bias, and improve the validity and reliability of the findings. The design allowed adjustment for gestational age, a major factor influencing coagulation parameters. The blood parameters were measured prospectively in the same laboratory in the third trimester of pregnancy (baseline) prior to labour and birth, and blood loss was measured at childbirth, addressing the risk of reverse causality. Another strength is reproducibility. We examined the relationship of Hb with five different parameters of coagulation and all suggested the same effect. We were also able to replicate the findings using haematocrit as the exposure variable.

The findings are generalizable to the population in India as data was collected from 10 hospitals across four states in India, which are different in terms of their socioeconomic contexts, healthcare facilities, food habits, prevalence of malnutrition and anaemia among pregnant women, and burden of maternal complications and death. The physiological changes associated with anaemia observed in our study are likely to be generalizable to all pregnant women, globally.

One limitation was the 12% loss to follow-up due to staff problems in one hospital. None of the participants in that hospital were followed up during or after childbirth, thus any bias due to loss to follow-up is likely to be minimal, as it was not related to the exposures or outcomes examined in the study. The mean Hb in women who were followed-up was not different from those who were not followed-up. Although we objectively measured blood loss at childbirth using a calibrated blood collection drape (for vaginal birth) and from suction bottle and soaked sponges (for caesarean birth), we cannot rule out measurement errors, but as mentioned earlier, the methods conformed to the recommendations of ACOG. In addition, there is no evidence that clinician estimated blood loss or blood loss measured by calibrated drape is associated with differential misclassification of PPH. Therefore, it is less likely that the results are influenced by the methods used for ascertaining PPH at childbirth. 1.6% of the study population had PPH which was comparable with the rate estimated in a previous study (1.1%)[34], but the lower number of events (n=19) reduced the statistical power of the analysis for the secondary objective, which we acknowledge as a limitation. Low number of events also limited the statistical power of the effect of the interaction between low Hb and high D-dimer on increased probability of PPH. Further, despite using standardised laboratory procedures and accounting for time taken for the blood samples to reach the national reference laboratory from the study hospitals, we cannot completely rule out measurement errors for the blood parameters.

Conclusion

In this study of pregnant women, measures of the coagulation parameters in the third trimester were significantly associated with the severity of anaemia. We identified a substantial independent effect of high INR and low Hb on increased risk of PPH at childbirth. Given the high prevalence of anaemia in pregnant women, globally, further studies are required to investigate the mechanisms through which coagulation parameters could increase the risk of PPH in pregnant women with anaemia.

For peer review only - http://bmjopen.bmj.com/site/about/guidelines.xhtml

Conflicts of interest: The authors declare that they have no competing interests.

Author statement: MN developed the concept and designed the study, conducted the statistical analysis, led the overall work as chief investigator, and wrote the first draft of the paper. SC, SSC, DD, GD, SDK, PK, PM, RM, AR, SR, IR, CSV, RKT, and FZ contributed equally, and their names are included in the alphabetic order of their last name. They are collaborators and investigators for the study, contributed to developing the study, and led the work in their respective institution. They also edited the paper. NK and AA contributed to developing the laboratory measures for the study, and AA edited the laboratory measurement section of the paper. CO provided statistical expertise, and contributed to writing the statistical methods and results. JA contributed to developing the concept for the study and edited the paper. SJS and MAL contributed to interpreting the results of the study and edited the paper. MQ provided statistical advice. CB, MK, JJK are advisors and have contributed to developing the study. JJK also edited the paper.

Ethics approval: The study was approved by the institutional review boards (IRB) of each coordinating Indian institution, namely: Srimanta Sankaradeva University of Health Sciences, Guwahati, Assam; Nazareth hospital, Shillong, Meghalaya; Emmanuel Hospital Association, New Delhi; Mahatma Gandhi Institute of Medical Sciences, Sevagram, Maharashtra; and the Institute of Medical Sciences, Banaras Hindu University, Varanasi, Uttar Pradesh. It also received approval from the Government of India’s Health Ministry’s Screening Committee, the Indian Council of Medical Research, New Delhi and by the Oxford Tropical Research Ethics Committee (OxTREC), University of Oxford, UK.

Funding: The study was funded by a Nuffield Department of Population Health (NDPH) Pump-priming award, and the MaatHRI platform is funded by a Medical Research Council Career Development Award to Manisha Nair (Grant Ref: MR/P022030/1). The funders had no role in the study design, data collection, analysis or writing of the report. Manisha Nair had full access to all the information for the paper and had final responsibility for the decision to submit for publication.

Data statement: The data and metadata used in this study are available for free and can be obtained by contacting the corresponding author.

Figure legends

Figure-1: Inverted J-shaped association between Hb and platelets

Figure-2: Relative difference in D-dimer across the categories of anaemia

Figure-3: Absolute difference in fibrinogen across the categories of anaemia

Figure-4: Relative difference in D-dimer/fibrinogen ratio across the categories of anaemia

Figure-5: Absolute difference in platelets across the categories of anaemia

Figure-6: Relative difference in INR across the categories of anaemia

Supporting information

Table-S1: Traceability of Assays

For peer review only - http://bmjopen.bmj.com/site/about/guidelines.xhtml

Table-S2: Assay Information and performance characteristics

Table-S3: Sample size calculations

Table-S4: Association of coagulation parameters with haematocrit at third trimester

Figure S1: Flow chart showing the study population

Figure S2: Predicted probability of PPH observed by fitting an interaction between Hb and D-dimer

REFERENCES

1. World Health Organisation. Nutritional anaemias: tools for effective prevention and control. Geneva: WHO, 2017.
2. Kavle JA, Stoltzfus RJ, Witter F, et al. Association between anaemia during pregnancy and blood loss at and after delivery among women with vaginal births in Pemba Island, Zanzibar, Tanzania. *Journal of health, population, and nutrition* 2008;26(2):232-40. [published Online First: 2008/08/09]
3. Nair M, Choudhury MK, Choudhury SS, et al. Association between maternal anaemia and pregnancy outcomes: a cohort study in Assam, India. *BMJ global health* 2016;1(1):e000026. doi: 10.1136/bmjgh-2015-000026 [published Online First: 2016/04/07]
4. Guignard J, Deneux-Tharaux C, Seco A, et al. Gestational anaemia and severe acute maternal morbidity: a population-based study. *Anaesthesia* 2020;n/a(n/a) doi: 10.1111/anae.15222
5. World Health Organisation. The global prevalence of anaemia in 2011. Geneva: WHO, 2015.
6. Pavord S, Daru J, Prasannan N, et al. UK guidelines on the management of iron deficiency in pregnancy. *British Journal of Haematology* 2020;188(6):819-30. doi: 10.1111/bjh.16221
7. Thornton P, Douglas J. Coagulation in pregnancy. *Best practice & research Clinical obstetrics & gynaecology* 2010;24(3):339-52. doi: 10.1016/j.bpobgyn.2009.11.010 [published Online First: 2010/01/26]
8. Réger B, Péterfalvi Á, Litter I, et al. Challenges in the evaluation of D-dimer and fibrinogen levels in pregnant women. *Thrombosis research* 2013;131(4):e183-e87. doi: https://doi.org/10.1016/j.thromres.2013.02.005
9. Katz D, Beilin Y. Disorders of coagulation in pregnancy. *BJA: British Journal of Anaesthesia* 2015;115(suppl_2):ii75-ii88. doi: 10.1093/bja/aev374
10. Szecsi PB, Jorgensen M, Klajnbard A, et al. Haemostatic reference intervals in pregnancy. *Thrombosis and haemostasis* 2010;103(4):718-27. doi: 10.1160/th09-10-0704 [published Online First: 2010/02/23]
11. van der Pol LM, Tromeur C, Bistervels IM, et al. Pregnancy-Adapted YEARS Algorithm for Diagnosis of Suspected Pulmonary Embolism. *New England Journal of Medicine* 2019;380(12):1139-49. doi: 10.1056/NEJMoa1813865
12. Nair M, Bezbaruah B, Bora A, et al. Maternal and perinatal Health Research Collaboration, India (MaatHRI): methodology for establishing a hospital-based research platform in a low and middle income country setting. *F1000Research* 2020
13. Hajsadeghi S, Kerman SR, Khojandi M, et al. Accuracy of D-dimer:fibrinogen ratio to diagnose pulmonary thromboembolism in patients admitted to intensive care units. *Cardiovasc J Afr* 2012;23(8):446-56. doi: 10.5830/CVJA-2012-041
14. Kara H, Bayir A, Degirmenci S, et al. D-dimer and D-dimer/fibrinogen ratio in predicting pulmonary embolism in patients evaluated in a hospital emergency department. *Acta clinica Belgica* 2014;69(4):240-5. doi: 10.1179/2295333714y.0000000029 [published Online First: 2014/07/12]

For peer review only - http://bmjopen.bmj.com/site/about/guidelines.xhtml

15. Sentilhes L, Winer N, Azria E, et al. Tranexamic Acid for the Prevention of Blood Loss after Vaginal Delivery. 2018;379(8):731-42. doi: 10.1056/NEJMoa1800942
16. Widmer M, Piaggio G, Nguyen TMH, et al. Heat-Stable Carbetocin versus Oxytocin to Prevent Hemorrhage after Vaginal Birth. 2018;379(8):743-52. doi: 10.1056/NEJMoa1805489
17. American College of Obstetricians and Gynecologists. Quantitative Blood Loss in Obstetric Hemorrhage. Washington, DC: ACOG, 2019.
18. Kline JA, Williams GW, Hernandez-Nino J. D-dimer concentrations in normal pregnancy: new diagnostic thresholds are needed. *Clinical chemistry* 2005;51(5):825-9. doi: 10.1373/clinchem.2004.044883 [published Online First: 2005/03/15]
19. Adler G, Duchinski T, Jasinska A, et al. Fibrinogen Fractions in the Third Trimester of Pregnancy and in Puerperium. *Thrombosis Research* 2000;97(6):405-10. doi: http://dx.doi.org/10.1016/S0049-3848(99)00190-5
20. Imai K, Keele L, Tingley D. A general approach to causal mediation analysis. *Psychological methods* 2010;15(4):309-34. doi: 10.1037/a0020761 [published Online First: 2010/10/20]
21. Malhotra M, Sharma JB, Batra S, et al. Maternal and perinatal outcome in varying degrees of anemia. *International journal of gynaecology and obstetrics: the official organ of the International Federation of Gynaecology and Obstetrics* 2002;79(2):93-100. doi: 10.1016/s0020-7292(02)00225-4 [published Online First: 2002/11/13]
22. Tang J, Lin Y, Mai H, et al. Meta-analysis of reference values of haemostatic markers during pregnancy and childbirth. *Taiwanese Journal of Obstetrics and Gynecology* 2019;58(1):29-35. doi: https://doi.org/10.1016/j.tjog.2018.11.004
23. Bolliger D, Szlam F, Levy JH, et al. Haemodilution-induced profibrinolytic state is mitigated by fresh-frozen plasma: implications for early haemostatic intervention in massive haemorrhage. *British journal of anaesthesia* 2010;104(3):318-25. doi: 10.1093/bja/aeq001 [published Online First: 2010/02/06]
24. Bolliger D, Szlam F, Molinaro RJ, et al. Finding the optimal concentration range for fibrinogen replacement after severe haemodilution: an in vitro model. *British journal of anaesthesia* 2009;102(6):793-9. doi: 10.1093/bja/aep098 [published Online First: 2009/05/08]
25. Siegel JE, Swami VK, Glenn P, et al. Effect (or lack of it) of severe anemia on PT and APTT results. *American journal of clinical pathology* 1998;110(1):106-10. doi: 10.1093/ajcp/110.1.106 [published Online First: 1998/07/14]
26. Antwi-Baffour S, Kyeremeh R, Annison L. Severity of Anaemia Has Corresponding Effects on Coagulation Parameters of Sickle Cell Disease Patients. *Diseases* 2019;7(4):59. doi: 10.3390/diseases7040059
27. Roeloffzen WWH, Kluin-Nelemans HC, Bosman L, et al. Effects of red blood cells on hemostasis. *Transfusion* 2010;50(7):1536-44. doi: 10.1111/j.1537-2995.2010.02586.x
28. Abildgaard U, Heimdal K. Pathogenesis of the syndrome of hemolysis, elevated liver enzymes, and low platelet count (HELLP): a review. *European Journal of Obstetrics & Gynecology and Reproductive Biology* 2013;166(2):117-23. doi: https://doi.org/10.1016/j.ejogrb.2012.09.026
29. Scharbert G, Wetzel L, Berlinger L, et al. Effect of anemia on coagulation and platelet function: a whole blood in vitro study. *Crit Care* 2011;15(Suppl 1):P445-P45. doi: 10.1186/cc9865 [published Online First: 2011/03/11]
30. Horne MK, 3rd, Cullinane AM, Merryman PK, et al. The effect of red blood cells on thrombin generation. *Br J Haematol* 2006;133(4):403-8. doi: 10.1111/j.1365-2141.2006.06047.x [published Online First: 2006/04/29]
31. Kadikoylu G, Yavasoglu I, Bolaman Z, et al. Platelet parameters in women with iron deficiency anemia. *J Natl Med Assoc* 2006;98(3):398-402.
32. Soto AF, Ford P, Mastoris J. Thrombocytosis in Iron Deficiency Anemia: What the Primary Care Physician Needs To Know. *Blood* 2006;108(11):3723-23. doi: 10.1182/blood.V108.11.3723.3723

For peer review only - http://bmjopen.bmj.com/site/about/guidelines.xhtml

33. Uijttewaal WS, Nijhof EJ, Bronkhorst PJ, et al. Near-wall excess of platelets induced by lateral migration of erythrocytes in flowing blood. *The American journal of physiology* 1993;264(4 Pt 2):H1239-44. doi: 10.1152/ajpheart.1993.264.4.H1239 [published Online First: 1993/04/01]
34. Nair M, Choudhury MK, Choudhury SS, et al. IndOSS-Assam: investigating the feasibility of introducing a simple maternal morbidity surveillance and research system in Assam, India. 2016;1(1):e000024. doi: 10.1136/bmjgh-2015-000024 %J BMJ Global Health

For peer review only

For peer review only - http://bmjopen.bmj.com/site/about/guidelines.xhtml

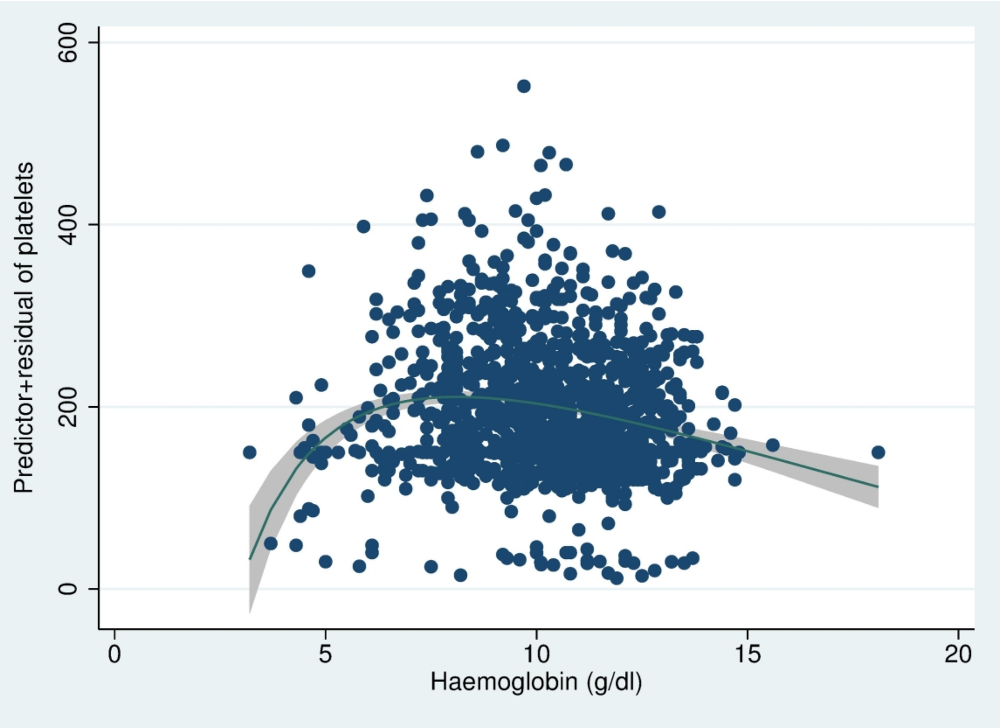

Figure-1: Inverted J-shaped association between Hb and platelets

139x101mm (600 x 600 DPI)

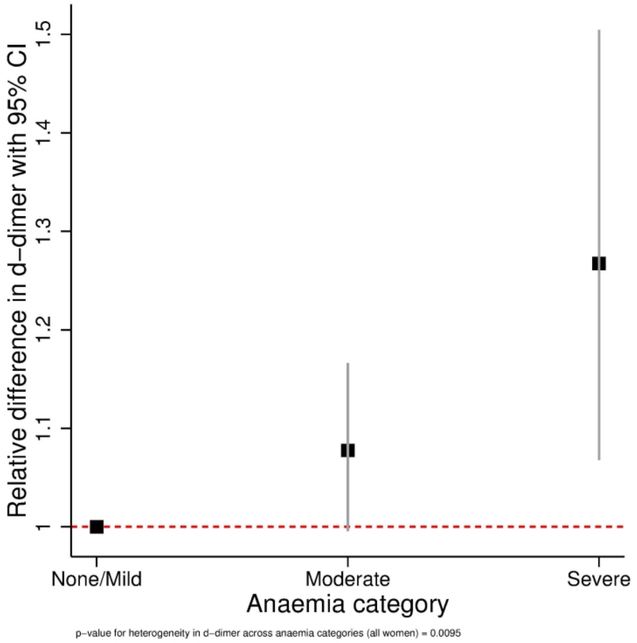

Figure-2: Relative difference in D-dimer across the categories of anaemia

139x101mm (600 x 600 DPI)

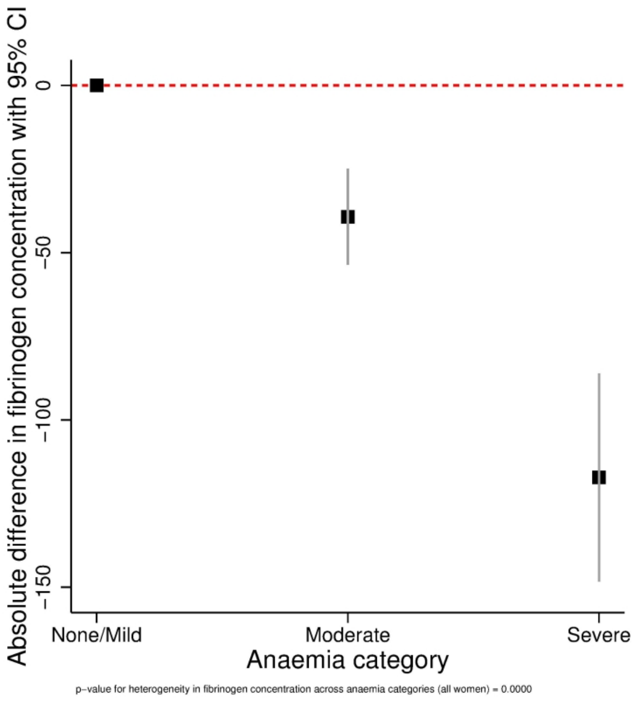

Figure-3: Absolute difference in fibrinogen across the categories of anaemia

139x101mm (600 x 600 DPI)

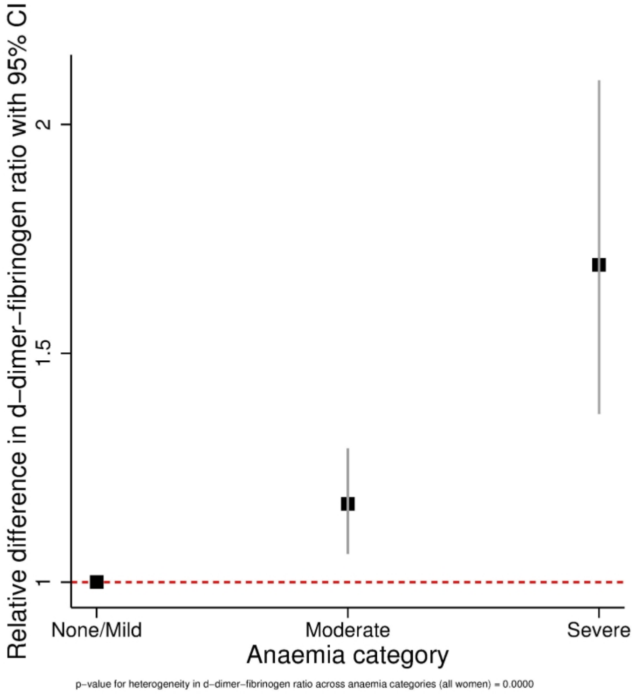

Figure-4: Relative difference in D-dimer/fibrinogen ratio across the categories of anaemia

139x101mm (600 x 600 DPI)

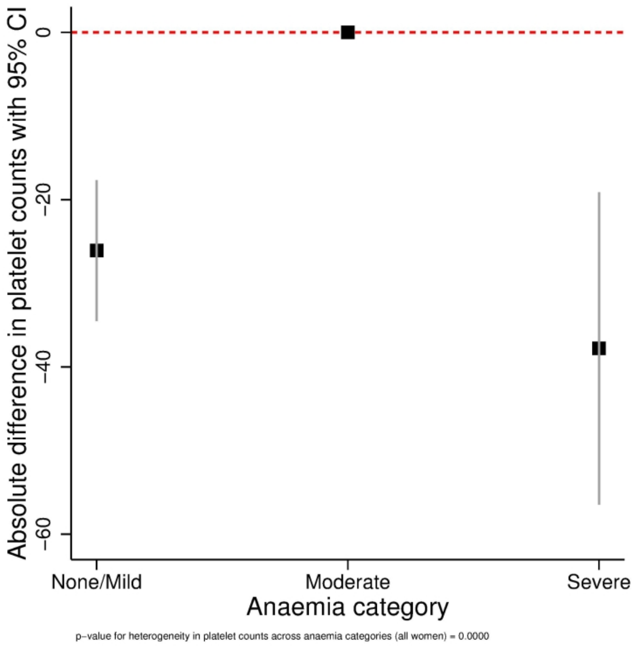

Figure-5: Absolute difference in platelets across the categories of anaemia

139x101mm (600 x 600 DPI)

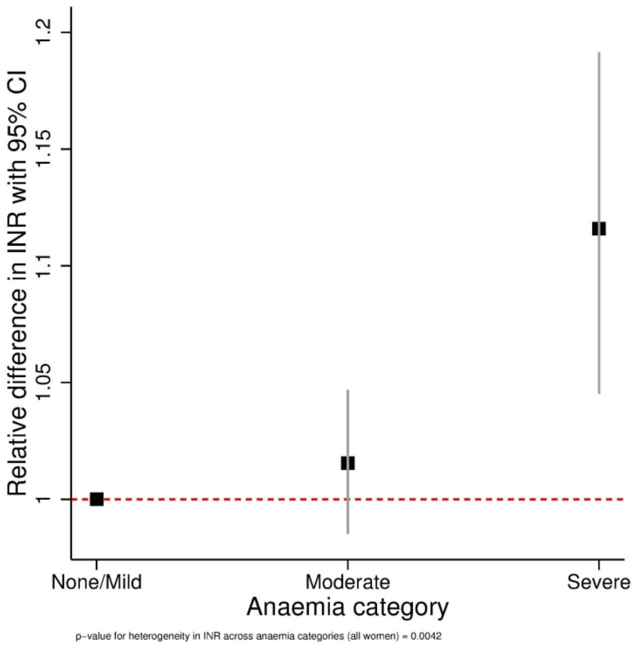

Figure-6: Relative difference in INR across the categories of anaemia

139x101mm (600 x 600 DPI)

Table-S1: Traceability of Assays

Sl No	Name of test	Calibrator traceability (reference material/ reference method)	Units	Typical Calibrator value	Calibrator uncertainty of measurement
1	Haemoglobin	1:250 dilution in NCCLS2 recommended reagent for the hemiglobincyanide (cyanmethemoglobin	g/dl	12.58	1.00%
2	Hematocrit	Calculated	%	calculated	NA
3	Platelets	A 1:101 dilution is made using a 20 µL TC pipette and 2 mL of 1% filtered ammonium oxalate (CLSI/ formerly NCCLS)	thou/mm3	214.1	6.00%
4	Serum Ferritin	WHO 3rd International Standard 94/572	ng/ml	Low 5.44<br>High 953	Low 19.5<br>High 9.3
5	Haemoglobin electrophoresis	NGSP Certification for A2/F	%	HbF-6.6 % and HbA2-6.7 %	HbF- Low- NA<br>High 1.8 %<br>HbA2- Low-NA, High- 3.6 %
6	D-Dimer	Pre-calibrated	mg/L FEU	NA	NA
7	Fibrinogen, Clotting activity	WHO	mg/dl	269	2.7%
8	International Normalized Ratio (INR)	Calculated			

NGSP - National Glycohemoglobin Standardization Program; CLSI – Clinical and Laboratory Standards Institute; HbF – Fetal haemoglobin; HbA2 - Haemoglobin Subunit Alpha 2; NA - Not applicable; WHO – World Health Organisation

For peer review only - http://bmjopen.bmj.com/site/about/guidelines.xhtml

Table-S2: Assay Information and performance characteristics

Sl No	Name of test	System used for the analysis	Method information (supplier/ method)	Manufacturers' Analytical Range	Laboratory Reportable range	Normal Reference range	Biological variation	Uncertainty of measurement	QC material	External Quality Assurance
1	Haemoglobin	DxH -800 (Beckman Coulter, Fullerton, CA, USA)	Photometric	0.0-99.9g/dl	1.25	13.-17.0g/dl (adult male)	2.1	4.0	Coulter 6c cell control	CAP
2	Hematocrit	DxH -800 (Beckman Coulter, Fullerton, CA, USA)	Automated calculation	0.99.9	NA	40-50% (adult male)	1.9	4.0	Coulter 6c cell control	CAP
3	Platelets	DxH -800 (Beckman Coulter, Fullerton, CA, USA)	Impedance/ Coulter principle	0.7000	10-1000	150-450thou/mm3 (adult male)	2.9	6.0	Coulter 6c cell control	CAP
4	Serum Ferritin	Siemens ADVIA Centaur	Chemiluminescence Immunoassay (CLIA)	0.5 – 1650 ng/ml	<0.5, >16500		14.2	22.5	BIO-RAD	CAP PT
5	Haemoglobin electrophoresis	Variant II Hemoglobin testing system (Bio-Rad, Hercules, CA, USA)	High Performance Liquid Chromatography	HbF-1.3-44.3 %<br>HbA2-1.6-18.7 %	HbF-1.3-99.8%<br>HbA2-1.6-18.7 %	HbF- <1.5 %<br>HbA2-1.5-3.5 %	HbF-5.6 %<br>HbA2-4.4 %	HbF-10.97 %<br>HbA2-8.62 %	BIO-RAD	CAP
6	D-Dimer	STA-R Evolution (Diagnostica Stago, Cedex, France)	Latex-enhanced immunoturbidimetry	0.22-20.0	0.22-20.0	< 0.50	10.4	20.38	Stago	CAP
7	Fibrinogen, Clotting activity	Sysmex CS5100 analyzer (Sysmex Corporation, Kobe, Japan)	Photo optical clot detection	30 -1400	50 -1200	200 - 400	13.8	27.05	Siemens	CAP
8	International Normalized Ratio (INR)	Calculated from Prothrombin time measured by Photo-optical clot Detection on Sysmex CS5100 analyzer (Sysmex Corporation, Kobe, Japan)	Calculated	Calculated	NA	0.9 – 1.1	NA	NA	Siemens	CAP

INR: International Normalized Ratio; NABL: National Accreditation Board for Testing and Calibration; CAP: College of American Pathologists; CAP PT - College of American Pathologists Proficiency Testing programme; HbF: Fetal haemoglobin; HbA2: Haemoglobin Subunit Alpha; QC: Quality Control; NA - Not applicable

For peer review only

For peer review only - http://bmjopen.bmj.com/site/about/guidelines.xhtml

Table-S3: Sample size calculations

Coagulation parameter	Concentration in general pregnant population Mean (SD)	Sample required for a range of expected difference in mean concentration of the blood parameters between the groups of anaemia in either direction					
		10% difference		20% difference		30% difference	
		n1	Total	n1	Total	n1	Total
D-Dimer (mg/dl)	0.11 (0.573)[7]	**514**	**1028**	129	258	58	116
Fibrinogen (mg/dl)	379 (0.78)[8]	90	180	23	46	10	20

n1 – women with haemoglobin concentration <10g/dl; n2 – women with haemoglobin concentration ≥10g/dl

Table-S4: Association of coagulation parameters with haematocrit at third trimester

Independent variables	Outcome variables	
	D-Dimer	
	Unadjusted coefficients (95% CI)	Adjusted* coefficients (95% CI)
Haematocrit (HCT)	0.99 (0.98 to 0.99)	0.99 (0.98 to 0.99)
HCT ≥30%	1 (ref)	1 (ref)
HCT <30%	1.13 (1.03 to 1.25)	1.13 (1.03 to 1.24)
	Fibrinogen	
	Unadjusted coefficients (95% CI)	Adjusted* coefficients (95% CI)
Haematocrit (HCT)	4.57 (3.37 to 5.77)	5.69 (4.51 to 6.88)
HCT ≥30%	0 (Ref)	0 (Ref)
HCT <30%	-58.3 (-75.4 to -41.1)	-68.1 (-84.8 to -51.3)
	D-Dimer/Fibrinogen ratio	
	Unadjusted coefficients (95% CI)	Adjusted* coefficients (95% CI)
Haematocrit (HCT)	0.98 (0.97 to 0.99)	0.97 (0.96 to 0.98)
HCT ≥30%	1 (ref)	1 (ref)
HCT <30%	1.35 (1.19 to 1.52)	1.39 (1.24 to 1.56)
	INR	
	Unadjusted coefficients (95% CI)	Adjusted* coefficients (95% CI)
Haematocrit (HCT)	0.99 (0.99 to 1.00)	0.99 (0.99 to 1.00)
HCT ≥30%	1 (ref)	1 (ref)
HCT <30%	1.02 (0.98 to 1.06)	1.05 (1.01 to 1.09)
	Platelets	
	Unadjusted coefficients (95% CI)	Adjusted* coefficients (95% CI)
Haematocrit (HCT)	-0.38 (-1.07 to 0.30)	-0.89 (-1.59 to -0.18)
HCT ≥30%	0 (ref)	0 (ref)
HCT <30%	4.84 (-4.93 to 14.61)	11.32 (1.42 to 21.23)

*Adjusted for gestational age, maternal age, PIH, pre-existing medical problems and hospital-code.

For peer review only - http://bmjopen.bmj.com/site/about/guidelines.xhtml

Figure-S1: Flow chart showing the study population

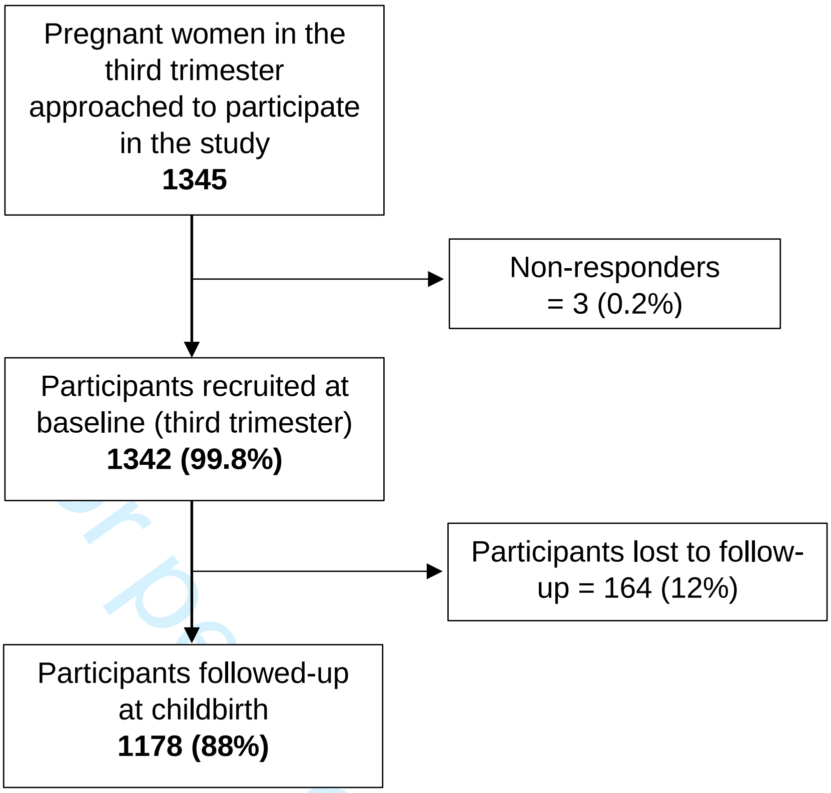

Figure S2: Predicted probability of PPH observed by fitting an interaction between Hb and D-dimer

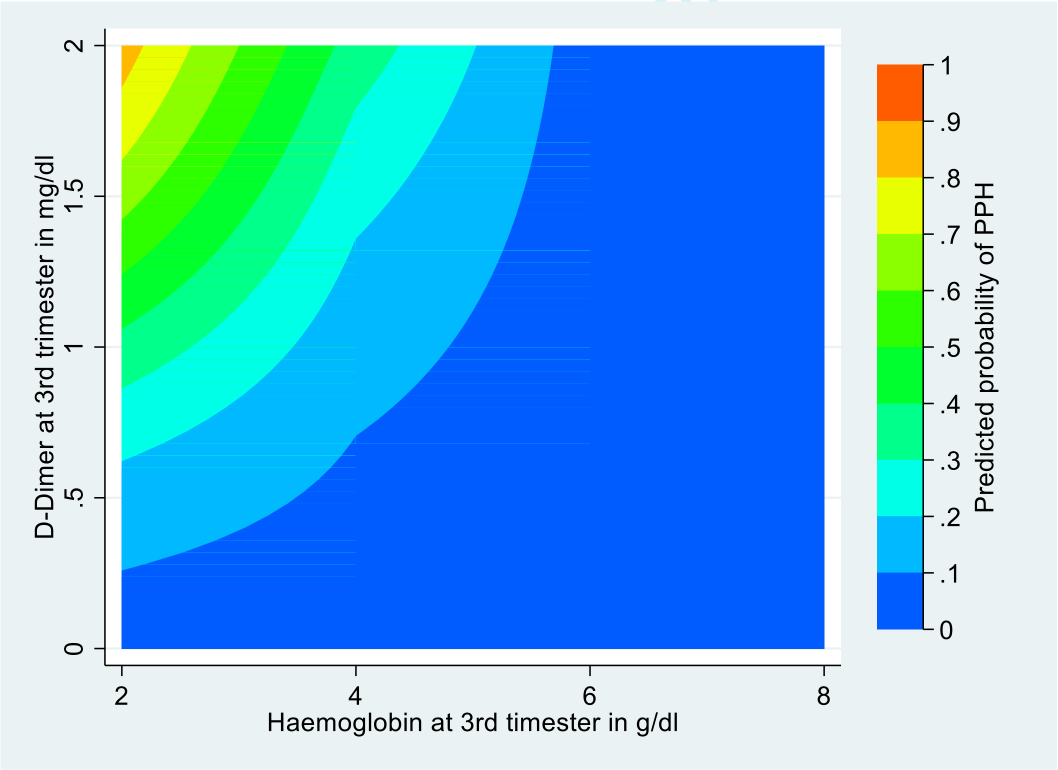

For peer review only - http://bmjopen.bmj.com/site/about/guidelines.xhtml

STROBE Statement—Checklist of items that should be included in reports of ***cohort studies***

	Item No	Recommendation	Page No
**Title and abstract**	1	(*a*) Indicate the study's design with a commonly used term in the title or the abstract	1
		(*b*) Provide in the abstract an informative and balanced summary of what was done and what was found	4
**Introduction**			
Background/rationale	2	Explain the scientific background and rationale for the investigation being reported	6
Objectives	3	State specific objectives, including any prespecified hypotheses	6
**Methods**			
Study design	4	Present key elements of study design early in the paper	6
Setting	5	Describe the setting, locations, and relevant dates, including periods of recruitment, exposure, follow-up, and data collection	6,7
Participants	6	(*a*) Give the eligibility criteria, and the sources and methods of selection of participants. Describe methods of follow-up	6,7
		(*b*) For matched studies, give matching criteria and number of exposed and unexposed	N/A
Variables	7	Clearly define all outcomes, exposures, predictors, potential confounders, and effect modifiers. Give diagnostic criteria, if applicable	6,7
Data sources/ measurement	8*	For each variable of interest, give sources of data and details of methods of assessment (measurement). Describe comparability of assessment methods if there is more than one group	6,7
Bias	9	Describe any efforts to address potential sources of bias	7,8
Study size	10	Explain how the study size was arrived at	8
Quantitative variables	11	Explain how quantitative variables were handled in the analyses. If applicable, describe which groupings were chosen and why	6,8
Statistical methods	12	(*a*) Describe all statistical methods, including those used to control for confounding	8
		(*b*) Describe any methods used to examine subgroups and interactions	
		(*c*) Explain how missing data were addressed	
		(*d*) If applicable, explain how loss to follow-up was addressed	
		(*e*) Describe any sensitivity analyses	
**Results**			
Participants	13*	(a) Report numbers of individuals at each stage of study—eg numbers potentially eligible, examined for eligibility, confirmed eligible, included in the study, completing follow-up, and analysed	9,10
		(b) Give reasons for non-participation at each stage	
		(c) Consider use of a flow diagram	
Descriptive data	14*	(a) Give characteristics of study participants (eg demographic, clinical, social) and information on exposures and potential confounders	9,10
		(b) Indicate number of participants with missing data for each variable of interest	
		(c) Summarise follow-up time (eg, average and total amount)	
Outcome data	15*	Report numbers of outcome events or summary measures over time	10

For peer review only

Main results	16	(*a*) Give unadjusted estimates and, if applicable, confounder-adjusted estimates and their precision (eg, 95% confidence interval). Make clear which confounders were adjusted for and why they were included<br>(*b*) Report category boundaries when continuous variables were categorized<br>(*c*) If relevant, consider translating estimates of relative risk into absolute risk for a meaningful time period	11, 12, 13, 14
Other analyses	17	Report other analyses done—eg analyses of subgroups and interactions, and sensitivity analyses	13
**Discussion**			
Key results	18	Summarise key results with reference to study objectives	14
Limitations	19	Discuss limitations of the study, taking into account sources of potential bias or imprecision. Discuss both direction and magnitude of any potential bias	16
Interpretation	20	Give a cautious overall interpretation of results considering objectives, limitations, multiplicity of analyses, results from similar studies, and other relevant evidence	14, 15
Generalisability	21	Discuss the generalisability (external validity) of the study results	16
**Other information**			
Funding	22	Give the source of funding and the role of the funders for the present study and, if applicable, for the original study on which the present article is based	19

*Give information separately for exposed and unexposed groups.

Note: An Explanation and Elaboration article discusses each checklist item and gives methodological background and published examples of transparent reporting. The STROBE checklist is best used in conjunction with this article (freely available on the Web sites of PLoS Medicine at http://www.plosmedicine.org/, Annals of Internal Medicine at http://www.annals.org/, and Epidemiology at http://www.epidem.com/). Information on the STROBE Initiative is available at http://www.strobe-statement.org.

For peer review only
